# MiRNA-449 family is epigenetically repressed and sensitizes to doxorubicin through ACSL4 downregulation in triple-negative breast cancer

**DOI:** 10.1038/s41420-024-02128-7

**Published:** 2024-08-22

**Authors:** Sandra Torres-Ruiz, Iris Garrido-Cano, Ana Lameirinhas, Octavio Burgués, Cristina Hernando, María Teresa Martínez, Federico Rojo, Begoña Bermejo, Marta Tapia, Juan Antonio Carbonell-Asins, Carlos Javier Peña, Ana Lluch, Juan Miguel Cejalvo, Eduardo Tormo, Pilar Eroles

**Affiliations:** 1 INCLIVA Biomedical Research Institute, Valencia, Spain; 2 Interuniversity Research Institute for Molecular Recognition and Technological Development (IDM), Universidad politécnica de Valencia, Universidad de Valencia, Valencia, Spain; 3 Bioengineering, Biomaterials and Nanomedicine Networking Biomedical Research Centre (CIBER-BBN), Madrid, Spain; 4 Department of Pathology, Hospital Clínico Universitario de València, Valencia, Spain; 5 Center for Biomedical Network Research on Cancer (CIBERONC), Madrid, Spain; 6 Department of Medical Oncology, Hospital Clínico Universitario de València, Valencia, Spain; 7 Department of Pathology, Fundación Jiménez Díaz, Madrid, Spain; 8 Department of Medicine, Universidad de Valencia, Valencia, Spain; 9 Department of Physiology, Universidad de Valencia, Valencia, Spain

**Keywords:** Breast cancer, Tumour biomarkers

## Abstract

Despite progress in breast cancer treatment, a significant portion of patients still relapse because of drug resistance. The involvement of microRNAs in cancer progression and chemotherapy response is well established. Therefore, this study aimed to elucidate the dysregulation of the microRNA-449 family (specifically, microRNA-449a, microRNA-449b-5p, and microRNA-449c-5p) and its impact on resistance to doxorubicin, a commonly used chemotherapeutic drug for the treatment of triple-negative breast cancer. We found that the microRNA-449 family is downregulated in triple-negative breast cancer and demonstrated its potential as a diagnostic biomarker. Besides, our findings indicate that the downregulation of the microRNA-449 family is mediated by the microRNAs-449/SIRT1-HDAC1 negative feedback loop. Moreover, it was found that the microRNA-449 family dysregulates the fatty acid metabolism by targeting *ACSL4*, which is a potential prognostic biomarker that mediates doxorubicin response through regulation of the drug extrusion pump ABCG2. Altogether, our results suggest that the microRNA-449 family might be a potential therapeutic target for the treatment of triple-negative breast cancer since it is implicated in doxorubicin response through ACSL4/ABCG2 axis regulation. Ultimately, our results also highlight the value of microRNAs-449 and ACSL4 as diagnostic and prognostic biomarkers in triple-negative breast cancer.

**Figure:**
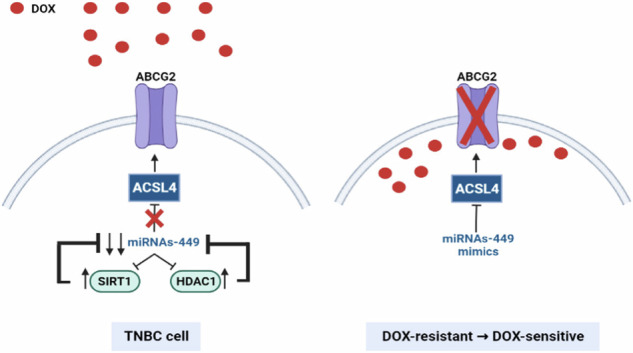
Proposed model of miRNAs-449 downregulation in TNBC and doxorubicin response. MiRNAs-449 are downregulated in TNBC through a negative feedback loop with SIRT1 and HDAC1. Moreover, ACSL4 increases ABCG2 expression, thus diminishing the intracellular doxorubicin concentration and promoting doxorubicin resistance. MiRNAs-449 overexpression downregulates the ACSL4/ABCG2 axis and sensitizes doxorubicin-resistant cells to doxorubicin. Created with BioRender. TNBC: triple-negative breast cancer; DOX: doxorubicin; SIRT1: Sirtuin 1; HDAC1: Histone deacetylase 1; ACSL4: Acyl-CoA Synthetase Long-Chain Family Member 4; ABCG2: ATP-binding cassette superfamily G member 2.

Proposed model of miRNAs-449 downregulation in TNBC and doxorubicin response. MiRNAs-449 are downregulated in TNBC through a negative feedback loop with SIRT1 and HDAC1. Moreover, ACSL4 increases ABCG2 expression, thus diminishing the intracellular doxorubicin concentration and promoting doxorubicin resistance. MiRNAs-449 overexpression downregulates the ACSL4/ABCG2 axis and sensitizes doxorubicin-resistant cells to doxorubicin. Created with BioRender. TNBC: triple-negative breast cancer; DOX: doxorubicin; SIRT1: Sirtuin 1; HDAC1: Histone deacetylase 1; ACSL4: Acyl-CoA Synthetase Long-Chain Family Member 4; ABCG2: ATP-binding cassette superfamily G member 2.

## Background

Breast cancer (BC) is the most diagnosed type of cancer and the leading cause of cancer-related death in women worldwide [1]. BC patients’ treatment options are based on the expression of progesterone receptor (PR), estrogen receptor (ER), and human epidermal growth factors receptor type 2 (HER2), among other factors. Hormone blockers and inhibitors are used in patients with hormone receptor-positive BC, while anti-HER2 drugs are administered to those patients with HER2-positive BC. These targeted therapies are commonly used in combination with chemotherapy. However, the triple-negative BC (TNBC) subtype is characterized by the lack of expression of these biomarkers, which makes chemotherapy the most eligible treatment option in clinical practice [2]. TNBC accounts for 15% of BC cases and is considered the most aggressive and invasive BC subtype, presenting the worst prognosis [3, 4]. Approximately 50% of TNBC patients relapse after receiving chemotherapy [5], which evidences the need for new therapeutic targets. In this scenario, adaptative chemoresistance, which refers to the preexistence of resistant tumor cells, is currently under study [6–9]. Concretely, doxorubicin is often included in neoadjuvant regimes in TNBC patients, and the presence of resistant tumor cells is known to limit its effectiveness [6, 10]. Therefore, it is crucial to decipher the molecular and genetic bases of drug resistance to find new therapeutic tools, which might help to improve the efficacy of current treatments.

In this sense, microRNAs (miRNAs) are found to be dysregulated in several cancer types by epigenetic reprogramming [11, 12], and emerged as potential prognostic molecules involved in biological processes and chemotherapy response [13–17], by either acting as tumor suppressors or tumor promoters. MiRNAs are small non-coding RNAs that bind by seed sequence complementarity to the 3’UTR region of their target mRNAs, leading to gene silencing by mRNA degradation or translational inhibition [18]. In particular, the miRNA-449 family (miRNA-449a, miRNA-449b-5p, and miRNA-449c-5p) of tumor suppressors is located in the second intron of the *CDC20B* gene [19], and has been reported to promote cell cycle arrest and apoptosis, as well as to inhibit cell proliferation, migration, and invasion in several cancer types [20–26].

This study aims to clarify the dysregulation of the miRNAs-449’s in TNBC and to understand its involvement in doxorubicin response.

## Results

### The miRNAs-449 are potential diagnostic biomarkers and their lower expression is associated with poor prognosis in TNBC

**Fig. 1:**
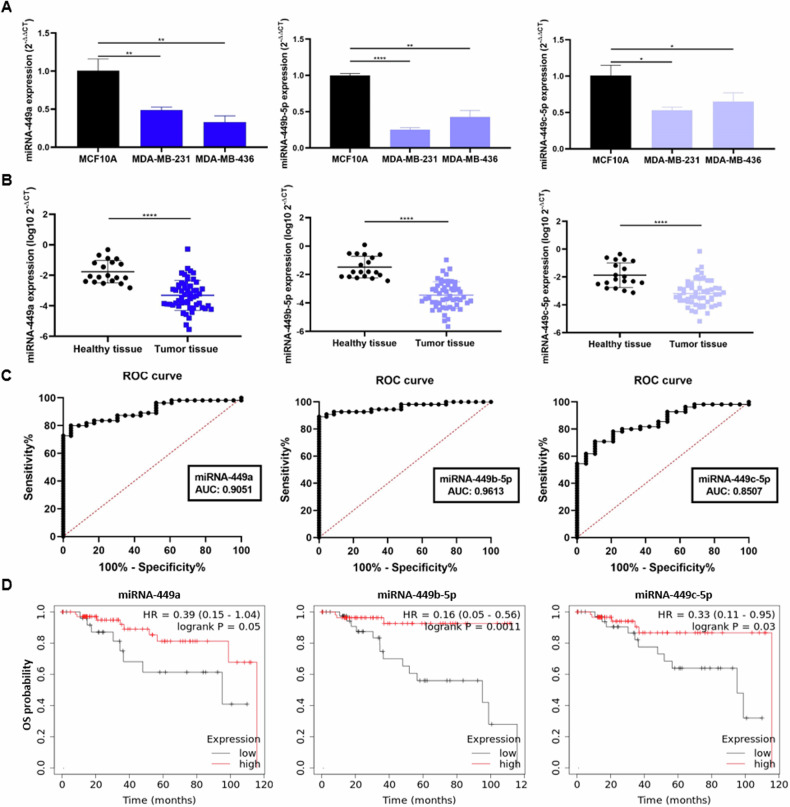
MiRNA-449 family downregulation is associated with worst OS and is a diagnostic biomarker for TNBC. **A** MiRNA-449 family (miRNA-449a, miRNA-449b-5p, and miRNA-449c-5p) expression was analyzed by RT-qPCR in TNBC cell lines (MDA-MB- 231 and MDA-MB-436), and the non-tumor immortalized line (MCF10A) (mean ± SD) (*n* = 3). **B**. MiRNA-449 family expression was analyzed by RT-qPCR in a discovery cohort of TN (*n* = 55) sample patients and healthy tissues (*n* = 19) samples (mean ± SD). **C** ROC curve analyses were performed for miRNAs-449 in TNBC tissue samples (*n* = 55) and healthy tissue samples (*n* = 19). **D** Representation of OS Kaplan Meier curves in TNBC samples (*n* = 97) from TCGA group based on an optimal cut-off of miRNAs-449 for low (black) and high (red) expression (*p* = 0.0500 for miRNA-449a, *p* = 0.0011 for miRNA-449b-5p, and *p* = 0.0300 for miRNA-449c-5p). *p < 0.05; **p < 0.01, ****p < 0.0001.

To elucidate the role of miRNAs-449 in TNBC, their basal expression levels were first evaluated by RT-qPCR in the TNBC cell lines MDA-MB-231 and MDA-MB-436 and in the non-tumoral cell line MCF10A. The results showed a significantly lower expression of miRNAs-449 in both TNBC cell lines compared to the MCF10A cell line (up to 0.8-fold decrease) (Fig. 1A). Similarly, the expression of these miRNAs was also evaluated by RT-qPCR in a cohort of primary biopsies from TNBC tissue samples (*n* = 55) and healthy breast tissue samples (*n* = 19). MiRNA-449 family was found significantly downregulated in TNBC tissue samples compared to healthy tissue samples (Fig. 1B), thus confirming the underexpression of these miRNAs not only in cell lines but also in TNBC patient samples.

To study the clinical relevance of these miRNAs, their value as diagnostic biomarkers was assessed by ROC curve analysis. We found that miRNAs-449 expression differed between TNBC patients and healthy tissue samples, with an AUC of 0.9051 for miRNA-449a, 0.9613 for miRNA-449b-5p, and 0.8507 for miRNA-449c-5p. Using the optimal cut-off value for miRNA-449a (−2.556), a sensitivity of 80% and specificity of 95.65% were obtained. Similarly, a sensitivity of 90.91% and specificity of 95.65% were detected for miRNA-449b-5p (best cut-off = −2.327), and a sensitivity of 61.82% and specificity of 94.74% were obtained for miRNA-449c-5p (best cut-off = −3.015) (Fig. 1C). Notably, the detection based on a combination signature of all miRNAs helped to discern between TNBC patient and healthy individual-derived tissue samples with an AUC of 0.9148, a specificity of 78.18%, and a sensitivity of 100% (optimal cut-off = 0.503) (Fig. S3).

The prognostic value was also evaluated in silico in 97 TNBC patients from the TCGA cohort. Poorer OS was found to be associated with lower miRNA-449a expression (HR = 0.39, 95% confidence Interval (CI) = 0.15–1.04 *p* = 0.0500), although it did not reach statistical significance. In addition, poorer OS was significantly associated with miRNA-449b-5p (HR = 0.16 CI = 0.05–0.56 *p* = 0.0011) and miRNA-449c-5p (HR = 0.33 CI = 0.11–0.95 *p* = 0.0300) (Fig. 1D).

### The miRNA-449 family expression is regulated by a negative feedback loop with HDAC1 and SIRT1

**Fig. 2:**
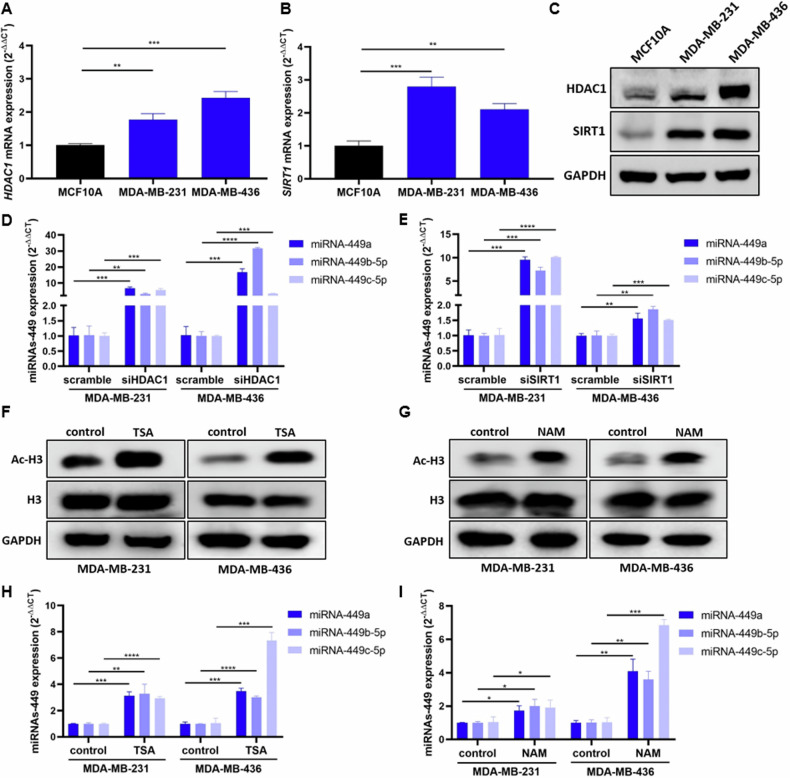
HDAC1 and SIRT1 are overexpressed in TNBC and inhibit miRNAs-449 expression by histone deacetylation. HDAC1 (**A**) and SIRT1 (**B**) expression were analyzed by RT-qPCR (mean ± SD) (*n* = 3)and Western blot (**C**) in TNBC cell lines (MDA-MB-231 and MDA-MB-436) and the non-tumor immortalized line MCF10A. MiRNA-449 family (miRNA-449a, miRNA-449b-5p, and miRNA-449c-5p) expression was analyzed by RT-qPCR in TNBC cell lines (MDA-MB- 231 and MDA-MB-436) after siHDAC1 (**D**) or siSIRT1 (**E**) transfection (mean ± SD) (*n* = 3). Acetyl-H3 (Ac-H3) and H3 expression was analyzed by Western blot in TNBC cell lines after TSA (**F**) (10 nM, 24 h) or NAM (**G**) (300 µM, 24 h) treatment. MiRNA-449 family expression was analyzed by RT-qPCR in TNBC cell lines after TSA (**H**) (10 nM, 24 h) or NAM (**I**) (300 µM, 24 h) treatment (mean ± SD) (*n* = 3). *p < 0.0500, **p < 0.01, ***p < 0.001, ****p < 0.0001.

Based on pre-existing literature on the regulation of miRNAs-449 expression by different epigenetic mechanisms [26–28], we assessed the involvement of histone acetylation modulation in TNBC. We evaluated the basal expression of two histone deacetylases, HDAC1 and SIRT1, in MDA-MB-231, MDA-MB-436, and MCF10A. The results showed a significant upregulation of HDAC1 and SIRT1 in TNBC cell lines compared to the MFC10A cell line at mRNA (up to 3-fold increase) (Fig. 2A, B) and protein level (Fig. 2C).

Given the histone deacetylase activity of HDAC1 and SIRT1, a possible implication of these two proteins in miRNAs-449 modulation was evaluated. *HDAC1* knockdown significantly induced miRNAs-449 overexpression in MDA-MB-231 and MDA-MB-436 cell lines (up to 31-fold increase compared to scramble) (Fig. 2D). Similarly, *SIRT1* knockdown also triggered the same effect in both TNBC cell lines (up to 10-fold increase compared to scramble control) (Fig. 2E). Pharmacological treatment with the histone deacetylase type I and II inhibitor (TSA) and SIRT1 inhibitor (NAM) was also performed. Both TSA and NAM treatment induced an increase of acetyl-H3 expression compared to control in both TNBC cell lines (Fig. 2F, G). Furthermore, TSA treatment significantly enhanced miRNAs-449 expression in both MDA-MB-231 and MDA-MB-436 cell lines (up to a 7-fold increase compared to non-treated control) (Fig. 2H). This upregulation was also observed after NAM treatment (up to a 7-fold increase compared to non-treated control) (Fig. 2I). Altogether, these results reinforced the hypothesis that miRNAs-449 expression is regulated by histone acetylation.

**Fig. 3:**
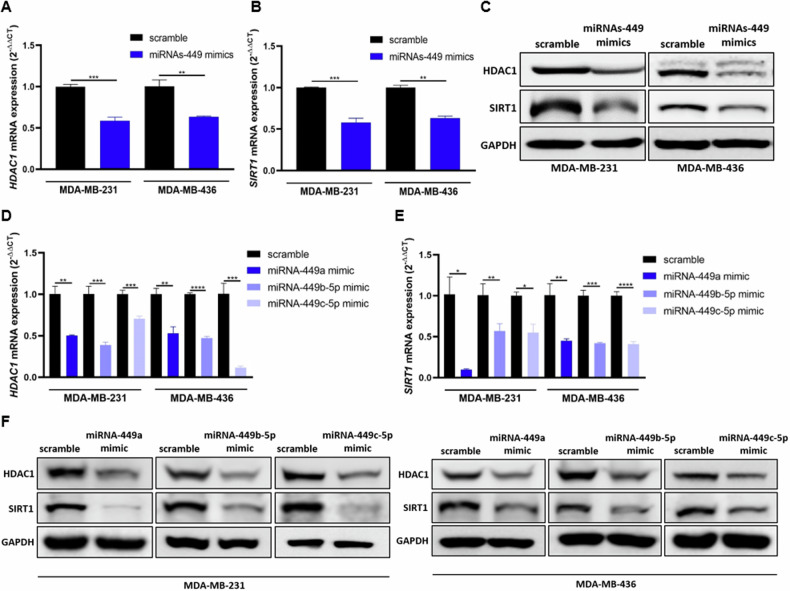
MiRNAs-449 overexpression downregulate HDAC1 and SIRT1 expression. MiRNAs-449 mimics transfection was performed in MDA-MB-231 and MDA-MB-436 cell lines. HDAC1 (**A**) and SIRT1 (**B**) expression was analyzed by RT-qPCR (mean ± SD) (*n* = 3) and Western blot (**C**). MiRNA-449a, miRNA-449b-5p, and miRNA-449c-5p mimics were transfected separately in MDA-MB-231 and MDA-MB-436 cell lines. HDAC1 (**D**) and SIRT1 (**E**) expression was analyzed by RT-qPCR (mean ± SD) (*n* = 3) and western blot (**F**). *p < 0.05, **p < 0.01, ***p < 0.001, ****p < 0.0001.

To assess if a feedback loop is regulating the expression of miRNAs-449 in BC, the expression of HDAC1 and SIRT1 was evaluated after miRNAs-449 overexpression. The results showed significant downregulation of HDAC1 and SIRT1 at the mRNA (up to 0.9-fold decrease compared to scramble) and protein levels upon miRNA-449a, miRNA-449b-5p, and miRNA-449c-5p co-overexpression or overexpression separately (Fig. 3A-F). In summary, our results support the existence of a miRNAs-449/SIRT1-HDAC1 negative feedback loop.

### The miRNA-449 family modulates fatty acid metabolism through *ACSL4* direct targeting

Hierarchical clustering of miRNAs-449 and cancer pathways using miRPath software predicted a significant interaction between these miRNAs and central carbon metabolism (*p* < 0.0001), fatty acid biosynthesis (*p* < 0.0001), and fatty acid metabolism (*p* < 0.0001) (Fig. S4), thus suggesting a fatty acid metabolism modulation by miRNA-449 family. MiRNAs-449 were predicted to target different genes involved in fatty acid biosynthesis by using TarBase v7.0: *Fatty Acid Synthase* (*FASN*), *ACSL4*, and *Acetyl-CoA carboxylase alpha* (*ACACA*). Based on previous literature, *ACSL4* is the only predicted target presenting a key role in TNBC having an inverse correlation with the expression of PR, ER, and HER2 [29–31]. These findings motivated further research focused on ACSL4.

**Fig. 4:**
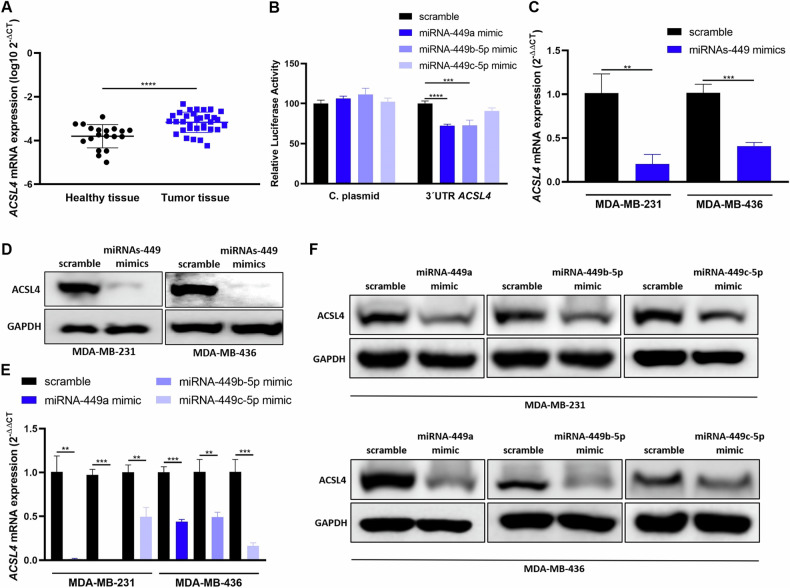
MiRNAs-449 downregulate ACSL4 expression by direct (miRNA-449a/b-5p) and indirect (miRNA-449c-5p) targeting. **A** *ACSL4* expression was analyzed by RT-qPCR in a discovery cohort of TNBC (*n* = 33) sample patients and healthy tissue samples (*n* = 19) (mean ± SD). **B** Luciferase reporter assay was performed in HEK-293T cell line co-transfected with pEZX-MT06 (3’UTR *ACSL4* containing or empty vector) and miRNA-449a, miRNA-449b-5p or miRNA-449c-5p mimics separately (mean ± SD) (*n* = 4). ACSL4 expression was analyzed by RT-qPCR (mean ± SD) (*n* = 3) (**C**) and western blot (**D**) after miRNAs-449 mimics transfection in MDA-MB-231 and MDA-MB-436 cell lines. ACSL4 expression was analyzed by RT-qPCR (mean ± SD) (*n* = 3) (**E**) and Western blot (**F**) after miRNAs-449 mimics transfection separately in MDA-MB-231 and MDA-MB-436 cell lines. **p < 0.01, ***p < 0.001, ****p < 0.0001. C plasmid: Control plasmid.

Thus, basal expression of *ACSL4* was analyzed in our cohort of primary biopsies from TNBC and healthy breast tissue samples, which showed a significantly higher expression in TNBC patient’s tissues than in healthy tissues (*p* < 0.0001) (Fig. 4A). These results suggest an inverse correlation with miRNAs-449 expression in TNBC.

To evaluate *ACSL4* as a potential target of miRNAs-449 in BC, the luciferase reporter gene assay was carried out. MiRNAs-449a and miRNA-449b-5p significantly decreased the luciferase activity when co-transfected with the 3′UTR-*ACSL4* containing plasmid (28% and 27% decrease for miRNA-449a and miRNA-449b-5p, respectively, compared to scramble control) but no effect was detected upon co-transfection with a control plasmid. This indicated that *ACSL4* 3′UTR is a direct target of miRNA-449a and miRNA-449b-5p but is not a direct target of miRNA-449c-5p (Fig. 4B). Additionally, ACSL4 expression was evaluated upon transfection with miRNAs-449 mimics, confirming that miRNAs-449 overexpression significantly downregulated ACSL4 at mRNA (up to 0.8-fold decrease compared to scramble control) and protein levels (Fig. 4C, D). Furthermore, the effect of each miRNA family member was separately assessed and showed significant downregulation of ACSL4 after overexpression of each miRNA (up to 0.98-fold decrease compared to scramble control) at mRNA and protein levels (Fig. 4E, F). These results suggest that ACSL4 is directly regulated by miRNA-449a and miRNA-449b-5p, and indirectly by miRNA-449c-5p.

### ACSL4 as a potential prognostic biomarker for chemotherapy response

**Fig. 5:**
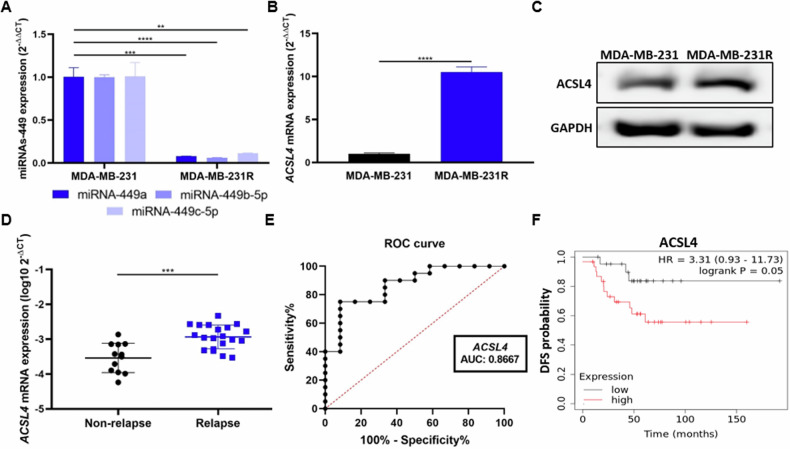
ACSL4 is overexpressed in MDA-MB-231R and patients who relapse after doxorubicin-containing chemotherapy treatment. **A** MiRNA-449 family (miRNA-449a, miRNA-449b-5p, and miRNA-449c-5p) expression was analyzed by RT-qPCR in MDA-MB-231 and MDA-MB-231R cell lines (mean ± SD) (*n* = 3). ACSL4 expression was analyzed by RT-qPCR (mean ± SD) (*n* = 3) (**B**) and Western blot (**C**) in MDA-MB-231 and MDA-MB-231R cell lines. **D** ACSL4 expression was analyzed by RT-qPCR in a discovery cohort of non-relapse (*n* = 12) and relapse (*n* = 20) TNBC patient tissue samples after chemotherapy treatment (mean ± SD). **E** ROC curve analysis was performed for ACSL4 in TNBC patient tissue samples who relapse (*n* = 20) or not (*n* = 12) after chemotherapy-containing treatment. **F** Representation of DFS Kaplan Meier curves in TNBC samples (*n* = 53) based on an optimal cut-off of ACSL4 for low (black) and high (red) expression (*p* = 0.0500). **p < 0.0100, ***p < 0.0010, ****p < 0.0001.

Further experiments were performed to analyze the biological role of miRNAs-449 in doxorubicin resistance through *ACSL4* modulation. First, basal expression of miRNAs-449 and ACSL4 was analyzed in doxorubicin-resistant (MDA-MB-231R) and doxorubicin-sensitive (MDA-MB-231) cell lines. The results showed a significantly lower expression of miRNAs-449 (up to 0.94-fold decrease) (Fig. 5A) and a higher expression of ACSL4 in doxorubicin-resistant cells, at mRNA (10-fold increase, *p* < 0.0001) and protein levels (Fig. 5B, C) when compared to the parental cell line.

To assess the clinical relevance of *ACSL4*, we analyzed its expression in naïve-tissue samples from a discovery cohort of TNBC patients posteriorly treated with chemotherapy. *ACSL4* was found significantly overexpressed in tumor samples from patients who experienced relapse (*n* = 20) when compared to non-relapse (*n* = 12) (*p* = 0.0003) (Fig. 5D). Next, the potential of *ACSL4* as a prognosis biomarker was assessed. *ACSL4* helped to discriminate between these two patient cohorts with an AUC of 0.8667, a sensitivity of 75%, and a specificity of 91.67% (optimal cut-off = −3.128) (Fig. 5E). The prognostic value of *ACSL4* was also predicted in a cohort of 53 TNBC patients in silico. Kaplan–Meier curves showed a lower DFS in TNBC patients with high expression of *ACSL4* compared to those with low *ACSL4* expression (HR = 3.31, CI = 0.93–11.73, *p* = 0.0500) (Fig. 5F), although it did not reach statistical significance. These results might indicate an involvement of *ACSL4* in chemotherapy response.

### MiRNAs-449 overexpression sensitizes to doxorubicin through ACSL4/ABCG2 axis downregulation

**Fig. 6:**
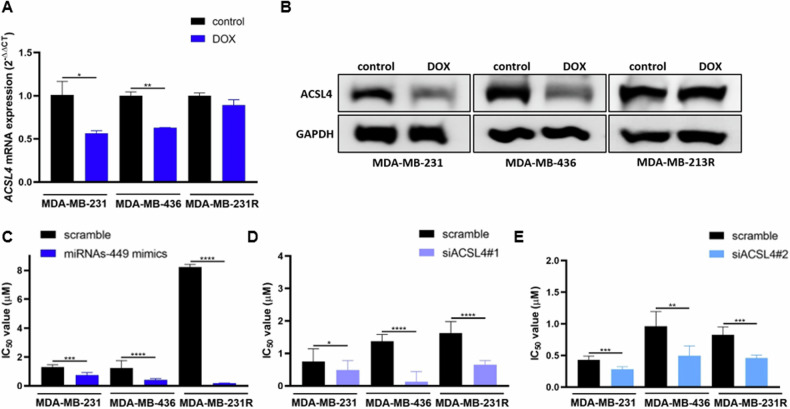
MiRNAs-449 overexpression sensitizes to doxorubicin through ACSL4 downregulation. ACSL4 expression was analyzed by RT-qPCR (mean ± SD) (*n* = 3) (**A**) and Western blot (**B**) in MDA-MB-231, MDA-MB-436, and MDA-MB-231R cell lines after doxorubicin treatment (1 µM, 48 h). Cell cytotoxicity was analyzed by WST-1 to determine the IC_50_ value (mean ± SD) (*n* = 3). MDA-MB-231, MDA-MB-436, and MDA-MB-231R cell lines were transfected with miRNAs-449 mimics (**C**), siACSL4#1 (**D**), or siACSL4#2 (**E**), and different doses of doxorubicin. *p < 0.0500, **p < 0.01, ***p < 0.001, ****p < 0.0001. DOX; doxorubicin.

In a previous study, we demonstrated that doxorubicin treatment induced overexpression of miRNAs-449 in doxorubicin-sensitive but not in doxorubicin-resistant TNBC cells [32]. These results were also confirmed in this study in MDA-MB-231 and MDA-MB-436 TNBC cell lines (up to 35-fold increase compared to control) (Fig. S5). Concordantly, *ACSL4* expression after doxorubicin treatment was found to be downregulated in doxorubicin-sensitive MDA-MB-231 (0.44-fold decrease compared to control, *p* = 0.0263) and MDA-MB-436 (0.37-fold decrease compared to control, *p* = 0.0015), but not in doxorubicin-resistant MDA-MB-231R cells (Fig. 6A, B).

To further explore the function of miRNAs-449 in the modulation of chemotherapy response, sensitivity to doxorubicin was evaluated after miRNAs-449 overexpression or *ACSL4* knockdown. MiRNAs-449 overexpression significantly decreased IC_50_ value (Fig. 6C). In line with this, IC_50_ values significantly decreased after *ACSL4* knockdown with two different siRNAs (Fig. 6D, E). These results suggest that the miRNA-449 family increases sensitivity to doxorubicin through *ASCL4* downregulation.

**Fig. 7:**
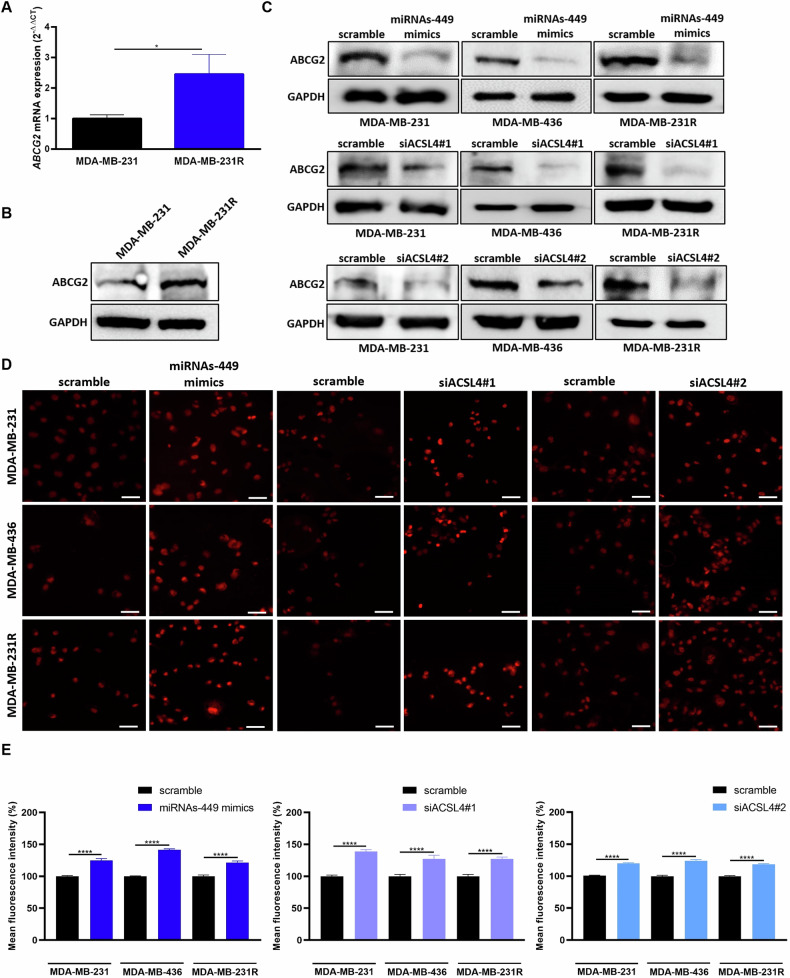
MiRNAs-449 overexpression and ACSL4 knockdown inhibit ABCG2 expression and increase the intracellular accumulation of doxorubicin. ABCG2 expression was analyzed by RT-qPCR (mean ± SD) (*n* = 3) (**A**) and Western blot (**B**) in MDA-MB-231 and MDA-MB-231R cell lines. **C** ABCG2 expression was analyzed by western blot in MDA-MB-231, MDA-MB-436, and MDA-MB-231R cell lines after miRNAs-449 mimics, siACSL4#1, or siACSL4#2 transfection. Representative images (**D**) (×40 magnification) and quantification of mean fluorescence intensity (**E**) of doxorubicin intracellular accumulation after miRNAs-449 mimics, siACSL4#1, or siACSL4#2 transfection, and doxorubicin treatment (5 µM, 3 h) (mean ± SEM). Scale bar: 50 µm. *p < 0.05, ****p < 0.0001.

Lastly, to elucidate the mechanism of action of miRNAs-449/ACSL4 axis modulation in doxorubicin response, the involvement of drug efflux was investigated. First, the expression of the drug transporter ABCG2 with affinity for doxorubicin was further explored and was found to be upregulated in doxorubicin-resistant compared to the parental cell line, both at mRNA (2.46-fold increase, *p* = 0.0183) and protein levels (Fig. 7A, B). Then, we evaluated the implication of miRNAs-449/ACSL4 axis in ABCG2 expression modulation. Luciferase reporter assay confirmed that there is no direct interaction between miRNAs-449 and *ABCG2* 3′UTR (Fig. S6). The indirect modulation was assessed in TNBC cell lines, which showed a downregulation of ABCG2 expression after miRNAs-449 overexpression or *ACSL4* knockdown (Fig. 7C), suggesting a negative and positive regulation of ABCG2 by miRNAs-449 and ACSL4, respectively. Subsequently, functional studies demonstrated that both miRNAs-449 overexpression and *ACSL4* knockdown significantly increased doxorubicin accumulation in MDA-MB-231 (25% increase compared to scrambled control), MDA-MB-436 (41% increase compared to scramble), and MDA-MB-231R cells (38% increase compared to scrambled control) (Fig. 7D, E). Overall, our findings suggest that overexpression of the miRNA-449 family decreases ABCG2 expression through ACSL4 downregulation, increasing the intracellular doxorubicin accumulation as a consequence.

## Discussion

Deciphering the molecular bases of TNBC is crucial for the design of new therapeutic approaches that will help to improve the efficacy of conventional chemotherapies such as doxorubicin. Gene expression studies pointed out the dysregulation of miRNAs in cancer and their functions as tumor suppressors or tumor promoters [33–37]. Specifically, this study focuses on the dysregulation of the miRNA-449 family and its role in the modulation of doxorubicin response.

We identified the miRNAs-449 to be downregulated in TNBC cell lines and patients. In addition, each member of the miRNA-449 family was able to discriminate between TNBC and healthy tissue samples, and the combination signature of miRNAs-449 was a stronger potential diagnostic biomarker with higher sensitivity and specificity. In line with these findings, previous studies pointed out the miRNAs-449 downregulation in several cancer types [21, 24–26], including TNBC [38–40]. Nevertheless, to our knowledge, no previous work evaluated the expression of miRNA-449c-5p, being our study the first one to analyze it. Lower miRNAs-449 expression was also associated with a worse prognosis, suggesting a tumor suppressor role. In the same trend, low miRNA-449a expression has been previously correlated with worse prognosis, advanced stages, and lymph node metastasis in non-small cell lung cancer, colorectal cancer, and BC, among others [24, 41, 42].

The miRNA-449 family downregulation might be partially explained by the epigenetic alteration of its promoter region. Concretely, our results showed an overexpression of the histone deacetylases HDAC1 and SIRT1. Hence, this explains lower miRNAs-449 expression levels in TNBC. HDAC1 and SIRT1 promote histone deacetylation by forming repressor complexes with transcription factors such as E2F1 [43, 44], which is a well-known miRNA-449 family activator [20]. It has been previously described the miRNA-449a upregulation upon *HDAC1* and *SIRT1* knockdown in colorectal cancer, and upon *HDAC1* knockdown in hepatocellular carcinoma [26, 27]. In this regard, HDAC chemical inhibition is also suggested to induce the overexpression of miRNA-449a, miRNA-449b-5p, and miRNA-449c-5p in hepatocellular carcinoma [28, 45], as well as the overexpression of miRNA-449a in BC and skeletal muscle cells [22, 45, 46]. In accordance with this, our results showed that genetic and pharmacological inhibition of HDAC1 and SIRT1 increased miRNAs-449 expression in TNBC cell lines. Moreover, HDAC1 and SIRT1 are directly regulated by miRNA-449a and miRNA-449b-5p [25, 47]. This *SIRT1* downregulation after miRNAs-449 overexpression was also elucidated in our previous study [32]. Additionally, our results pointed out an indirect downregulation of HDAC1 and SIRT1 by miRNA-449c-5p, altogether providing evidence of a negative regulatory feedback loop between the miRNAs-449 and HDAC1/SIRT1.

Considering the downregulation of miRNAs-449 in TNBC, artificial overexpression of this miRNA family can be proposed as a possible approach to revert the tumoral phenotype. However, its mechanism of action is still controversial. To elucidate the involvement of miRNAs-449 in the modulation of cancer pathways, bioinformatic analyses were performed and fatty acid metabolism was predicted to be regulated by this family of miRNAs. This is consistent with the fact that cancer cells have an altered lipid metabolism to support tumorigenesis and cancer progression [48]. Several studies showed that increased lipogenesis in cancer is due to the increased activity of enzymes involved in fatty acid activation, which is a prerequisite for the use of fatty acids in lipid metabolic pathways [49]. In line with this, bioinformatic analyses predicted fatty acid metabolism interaction through *FASN, ACACA*, and *ACSL4* genes. FASN and ACACA are known to be positively correlated with HER2-overexpressing tumors [50–52], while ACSL4 is highly expressed in ER, PR, HER2, and androgen receptor-negative tumors [29–31]. Based on this evidence, the current study focused on the relationship between miRNAs-449 and ACSL4 in TNBC.

ACSL4 is an enzyme that catalyzes the activation of long-chain fatty acids by esterification with coenzyme A, with arachidonic acid (AA) and eicosapentaenoic acid as substrate preferences [53]. Previous studies confirmed its association with aggressiveness in BC [31, 54–56]. Our results proved that miRNA-449a and miRNA-449b-5p directly inhibit ACSL4 expression, an interaction that has not been previously studied, as far as we know. Despite the lack of direct interaction between miRNA-449c-5p and *ACSL4* 3′UTR, we confirmed that overexpression of the miRNA inhibits ACSL4 expression in TNBC cells, which can be attributable to indirect mechanisms. Our results agree with other studies that revealed different binding specificities between miRNA-449a, miRNA-449b-5p, and miRNA-449c-5p [28].

Several studies suggest that miRNAs-449 act as tumor suppressors in different cancer types [20, 57, 58], including BC [38, 59], but its involvement in treatment resistance in the context of BC remains unknown. Furthermore, all previous studies focus only on miRNA-449a, whose overexpression is known to sensitize to ionizing radiation in prostate and lung cancer, to cisplatin in ovarian cancer, and tamoxifen and olaparib in BC [60–64]. In our previous study, we observed that E2F1 significantly upregulates miRNAs-449, which initiates a negative feedback loop that inhibits E2F1 activity by targeting CDK6 and CDC25A through Rb phosphorylation in TNBC doxorubicin-sensitive cells. This feedback mechanism ensures controlled E2F1-induced proliferation. However, miRNAS-449 were not upregulated after doxorubicin treatment in doxorubicin-resistant cells, thus promoting cell cycle progression. In this context, miRNAs-449 overexpression sensitized to doxorubicin [32]. Herein, we focused on exploring the role of miRNAs-449 in the modulation of doxorubicin response through *ACSL4* targeting. Regarding ACSL4, its knockdown has been associated with cisplatin, paclitaxel, and doxorubicin sensitivity in TNBC, and docetaxel sensitivity in prostate cancer [65–67]. In the same trend, *ACSL4* overexpression increased etoposide and tamoxifen resistance in the MCF7 cell line and lapatinib resistance in the SKBR3 cell line [31].

In this study, we observed that doxorubicin treatment increased the expression of the miRNA-449 family and decreased ACSL4 expression in the doxorubicin-sensitive cell line, but not in the doxorubicin-resistant cell line. The miRNAs-449 modulation in the doxorubicin-resistant model was also elucidated in our previous work [32], being the ACSL4 modulation the novelty of the current research. In addition, miRNAs-449 were found to be downregulated in doxorubicin-resistant cells, whereas ACSL4 was found to be overexpressed in both doxorubicin-resistant cells and in tumor samples from patients who relapsed after receiving chemotherapy, which was also associated with a worse DFS in TNBC patients. Moreover, the expression of *ACSL4* was able to discriminate between patients who either relapsed or not after chemotherapy, thus suggesting its role as a biomarker with potential prognostic value. However, the prognostic role of *ACSL4* in previous literature is debatable. On one hand, there are some studies supporting our outcomes, as higher *ACSL4* expression has been related to lower OS, DFS, and advanced stages of hepatocellular carcinoma. Furthermore, higher proliferation rates, migration capacity, and colony formation have been observed upon *ACSL4* overexpression in hepatocellular carcinoma and BC [65, 68]. Contrarily, other studies correlate higher *ACSL4* expression with better OS, RFS, and DFS, in lung adenocarcinoma, renal cell carcinoma, and colorectal cancer, among others [69–72], which could be associated with a tissue-specific role of *ACSL4*. Based on our results, this study suggests an oncogenic role for *ACSL4* in TNBC.

Both miRNAs-449 overexpression and *ACSL4* knockdown decreased doxorubicin IC_50_ value. These results suggest that overexpression of miRNAs-449 sensitizes TNBC cells to doxorubicin through *ACSL4* downregulation. The role of ACSL4 and its connection to fatty acid metabolism has been extensively studied, although its role in chemotherapy resistance is not as evident. Therefore, we aimed to elucidate this mechanism of action. In this context, ACSL4 overexpression is suggested to positively regulate the expression of drug extrusion pumps such as the ABC subfamily C members 8 (ABCC8) and 4 (ABCC4), and ABCG2, being ABCG2 modulation greater implicated in doxorubicin resistance. Particularly, ACSL4 overexpression increased ABCG2 expression through mTORC pathway modulation in BC [67]. Due to the high impact of this drug extrusion pump in BC, we focused on its study. ABCG2 is one of the most well-known molecules involved in drug resistance since it mediates the efflux of several drugs including doxorubicin [73, 74], and is overexpressed in different pathophysiological mechanisms such as cancer progression and drug resistance [75]. Our results pointed out for the first time a drug extrusion pump modulation by miRNAs-449. Concretely, we observed that miRNAs-449 overexpression negatively modulated ABCG2 expression through ACSL4 downregulation, thus increasing doxorubicin accumulation and sensitivity.

## Conclusions

In summary, our findings confirmed that the miRNA-449 family is downregulated in TNBC and might be useful as diagnostic biomarkers that are associated with poor prognosis. Our results evidence the regulation of miRNAs-449 expression through HDAC1 and SIRT1 histone deacetylases. Besides, in silico analysis revealed the significant involvement of miRNAs-449 in fatty acid metabolism by targeting *ACSL4*, which is a potential prognostic biomarker. We confirmed that miRNAs-449 inhibit ACSL4 expression, particularly through direct interactions involving miRNA-449a and miRNA-449b-5p. Moreover, miRNAs-449 were found to enhance doxorubicin sensitivity by downregulating ACSL4/ABCG2, leading to increased intracellular drug accumulation. Altogether, these findings suggest that miRNAs alone or in combination with ACSL4 inhibitors are a potential therapeutic strategy to overcome doxorubicin resistance.

## Materials and methods

### Cell lines and reagents

TNBC (MDA-MB-231 and MDA-MB-436), non-tumorigenic cell line (MCF10A), and human embryonic kidney 293T (HEK-293T) cell lines were used in this study. All cell lines were obtained from American Type Culture Collection (ATCC, Manassas, VA, USA) and grown in DMEM/F12 supplemented with 10% (v/v) of heat-inactivated fetal bovine serum (FBS, Gibco, Carlsbad, USA), 1% (v/v) penicillin-streptomycin (Biowest, Nuaillé, France) and 1% (v/v) l-glutamine 200 mM (Biowest) in a humidified atmosphere at 37 °C and 5% CO_2_. Federico Rojo group from the *Fundación Jiménez Díaz* (Madrid) kindly provided the TNBC cell line with acquired resistance to doxorubicin (MDA-MB-231R). Briefly, the MDA-MB-231R cell line was generated by exposing the MDA-MB-231 parental cell line to increased concentrations of doxorubicin (Pfizer, New York, NY, USA). Doxorubicin resistance maintenance was verified periodically by WST-1 cell viability assay by comparing doxorubicin IC_50_ value from MDA-MB-231R and MDA-MB-231 (Fig. S1). The cell lines used in this study were cultured at cell passages of less than 30 in order to maintain the viability and genetic stability of the cells, and routinely test for mycoplasma by using MycoStrip^TM^ 50 kit (#rep-mysnc-50, InvivoGen, Toulouse, France).

Cell lines were treated with 10 nM of trichostatin A (TSA, #T8551, Sigma-Aldrich, St. Louis, MO, USA) for 24 h or 300 µM of nicotinamide (NAM, #N0636, Sigma-Aldrich) for 24 h before real-time quantitative PCR (RT-qPCR) and Western blot analyses for epigenetic studies. DMSO at 0.2% and distilled water at 0.12% were used as negative controls, respectively.

Cells were treated with doxorubicin at 1 µM for 48 h for RT-qPCR and Western blot, 5 µM for 3 h for quantification of intracellular doxorubicin uptake, or a range of different doses (100, 50, 10, 5, 2.5, 1, 0.5, 0.25, 0.1, 0.01, 0.001 µM) for 48 h for further IC_50_ value analyses.

### Cell transfection

Cell lines were transfected with 100 nM of small interfering RNA (siRNA) targeting *Acyl-CoA Synthetase Long-Chain Family Member 4* (*ACSL4)* (siACSL4#1: #122222 and siACSL4#2: #122223, Ambion, Austin, TX, USA), *Sirtuin 1* (*SIRT1)* (#136457, Ambion), *Histone deacetylase 1* (*HDAC1)* (#120419, Ambion), or 50 nM of miRNAs-449 mimics (hsa-miRNA-449a (#MC11127), hsa-miRNA-449b-5p (#MC11521), and hsa-miRNA-449c-5p (#MC15616), Ambion). Scrambled sequences of miRNA (#4464059, Ambion) and siRNA (#4390844, Ambion) molecules were used as negative transfection controls.

Lipofectamine 2000 reagent (Invitrogen, Carlsbad, CA, USA) was used for transfection following the manufacturer’s instructions, and the medium was refreshed with a supplemented medium after 4 h. Transfection efficiency was verified after 48 h and 72 h by RT-qPCR and Western blot, respectively (Fig. S2).

### Luciferase reporter assay

The putative miRNAs-449 binding sites at the 3′UTR *ACSL4* mRNA (NM_004458.2) or 3′UTR *ABCG2* mRNA (NM_004827.2) were cloned into the pEZX-MT06 plasmid (Genecopeia, Guangzhou, China). pEZX-MT06 empty vector was used as a negative control plasmid. HEK-293T cells were seeded in a 24-well plate at 10^5^ cells/well and co-transfected with 5 ng/µl of 3′UTR containing plasmid or control plasmid pEZX-MT06, and 100 nM of miRNA-449a (#MC11127, Ambion), miRNA-449b-5p (#MC11521, Ambion), miRNA-449c-5p (#MC15616, Ambion) mimic or miRNA-negative transfection control (#4464059, Ambion) using lipofectamine 2000 reagent (Invitrogen) and following manufacturer’s instruction. Twenty-four hours post-transfection, luciferase activity was measured using Luc-Pair^TM^ Duo-Luciferase Assay Kit 2.0 (#217LF002, Genecopeia, Guangzhou, China) according to manufacturer’s recommendations, and luminescence was detected in the microplate reader LUMIstar Omega (BMG Labtech, Ortenberg, Germany).

### RT-qPCR

Total RNA, including miRNA, was extracted using TRIZOL reagent (Invitrogen) as described by the manufacturer. First, 1 µg of RNA was either retrotranscribed using a High-Capacity cDNA Reverse Transcription Kit (#4368813, Applied Biosystems, Waltham, MA, USA) for mRNA, or Taqman^TM^ MicroRNA Reverse Transcription Kit (#4366597, Applied Biosystems) for miRNAs following manufacturer’s protocol. RNU43 (#001608) and miRNAs-449 specific primers (miRNA-449a: #001030, miRNA-449b-5p: #001608, and miRNA-449c-5p: #001608) obtained from Applied Biosystems were used to generate cDNA from miRNA. RNA was then retro-transcribed to cDNA at either 25 °C for 10 min and 37 °C for 2 h for mRNA, or 16 °C for 30 min, 42 °C for 30 min, and 85 °C for 5 min for miRNA. The resulting cDNA was amplified using TaqMan^®^ Universal Master Mix (#M3004E, Applied Biosystems) and TaqMan 20× assays (Applied Biosystems) (miRNA-449a: #001030, miRNA-449b-5p: #001608, miRNA-449c-5p: 2410086_mat, *HDAC1*: #Hs02621185_s1, *SIRT1*: #Hs01009006_m1, *ACSL4*: #Hs00244871_m1) following manufacturer’s instructions on 9700HT Fast Real-Time PCR system (Applied Biosystems). PCR conditions were as follows: 50 °C for 2 min, 95 °C for 10 min, 40 cycles of 95 °C for 15 s, and 60 °C for 1 min. Data was analyzed following the comparative critical threshold (2^−ΔΔCT^) method using *GAPDH* (#Hs03929097_g1) or *RNU43*(#001095) as endogenous controls for mRNA and miRNA expression, respectively.

### Western blot

Cells were collected and lysed on ice using Pierce® RIPA buffer (#89900, Thermo Fisher Scientific) supplemented with a protease and phosphatase inhibitor cocktail (#A32961, Thermo Fisher Scientific) according to the manufacturer’s instructions. Cell lysates were then sonicated by the Sonics Vibra Cell VC 505 (Sonics&Materials, Newtown, MA, USA) (40% pulse and 10 s) and centrifuged (15 min, 13,200 rpm, 4 °C). Proteins were collected and quantified using the Pierce^TM^ BCA Protein Assay Kit (#23227, Thermo Fisher Scientific) following the manufacturer’s protocol. Protein samples (30 µg) were loaded in 6-12% sodium dodecyl sulfate (SDS)-polyacrylamide gels and transferred to nitrocellulose membranes (#1620115, BIO-RAD, Hercules, CA, USA). Membranes were blocked for 1 h with 5% of bovine serum albumin in 0.1% TBS-Tween20 and incubated with antibodies against SIRT1 (1:1000, #MA5-15677, Invitrogen), HDAC1 (1:1000, #PA1-860, Invitrogen), acetyl-H3 (1:1000, #ab47915, Abcam, Cambridge, UK), total H3 (1:1000, #9715S, Cell Signaling, Danvers, MA, USA), ACSL4 (1:1000, #PA5-27137, Invitrogen) or ABCG2 (1:1000, #4477S, Cell Signaling) at 4 °C overnight. GAPDH (1:2000, #MA5-15738, Thermo Scientific) was used as a loading control. The following day, membranes were washed with 0.1% TBS-Tween20 and incubated for 1 h with an anti-mouse (1:2000, #7076S, Cell Signaling) or anti-rabbit (1:2000, #7074S, Cell Signaling) IgG horseradish peroxidase-linked secondary antibody. After incubation, membranes were washed and signals were developed using Pierce^TM^ ECL Western blotting reagent (#32106, Thermo Fisher Scientific) or ultra-sensitive ECL Super Signal West Femto (#34095, Thermo Fisher Scientific), according to the manufacturer’s instructions, in the ImageQuant Las 4000 system (GE-Healthcare Bioscience, Chicago, IL, USA). Chemiluminescent images were analyzed by employing ImageJ-win64 for Windows.

### Cell viability assay

Twenty-four hours after transfection, cells were seeded at 10^4^ cells/well in a 96-well plate. The next day, cells were exposed to different concentrations of doxorubicin (from 0.001 to 100 µM) for 48 h. Viability was then measured with Colorimetric Cell Viability Kit II (WST-1) (#K304-2500, Deltaclon, Madrid, Spain), as described by the manufacturer. Absorbance was then measured at 450 nm and 650 nm (background) in the microplate reader Spectra Max Plus (Thermo Fisher Scientific).

### Quantification of intracellular doxorubicin uptake

Intracellular doxorubicin uptake was analyzed by confocal microscopy. Forty-eight hours post-transfection, 5 × 10^4^ cells/well were seeded in an 8-well chamber (#30108, SPL Life Sciences, Gyeonggi-do, South Korea). After 24 h, cells were exposed to 5 µM doxorubicin for 3 h. Then, cells were fixed with 4% paraformaldehyde (VWR BDH Chemicals, Matsonford, PA, USA) for 10 min, counterstained with 4′,6-diamidino-2-phenylindole (DAPI) (1:500 in PBS, Merck, Darmstadt, Germany) for 5 min, and mounted with glycerol (1:1 in PBS, Sigma-Aldrich). Fifteen pictures per well were acquired at the Central Medicine Research Unit (UCIM-UV) using a Leica DMi8 inverted fluorescence microscope (Wetzlar, Germany) with a PE4000 LED light source and DFC9000GT camera at 40x magnification. Doxorubicin and DAPI fluorescence were excited at 550 nm and 365 nm, and the emission was 590 nm and 435–485 nm, respectively. Mean doxorubicin fluorescence intensity per cell was analyzed with ImageJ (v1.53t, NIH, Bethesda, USA) for windows in a minimum of 150 cells for each condition, and results were normalized to scramble control condition. The analysis settings were the same for all pictures.

### In silico analyses

Kaplan–Meier plotter software (https://kmplot.com/analysis/) was used to evaluate the prognostic value of hsa-miRNA-449a, hsa-miRNA-449b-5p, hsa-miRNA-449c-5p, and *ACSL4*. For miRNAs analysis, overall survival (OS) was analyzed in TNBC patients from the TCGA dataset (*n* = 97) with auto-selected best cut-off. For *ACSL4* analysis, disease-free survival (DFS) was analyzed in TNBC patients (*n* = 53) with auto-selected best cut-off. The auto-selected best cut-off is described as previously [76]. The Hazard ratio (HR), log-rank *p-value*, and corresponding curves were calculated and plotted by the software.

DIANA TOOLS- mirPath v.3 (http://microrna.gr/miRPathv3) biological database was used to study cancer pathways affected by the miRNA-449 family while predicting possible targets. TarBase v7.0 was selected for miRNAs-449 target gene analyses, and pathways union was selected for regulated KEGG pathways analysis. Pathways union derives a fused *p-value* for each pathway by combining the previously calculated significance levels between each miRNA and each pathway, using Fisher’s Exact Test statistical analysis method.

### Patient samples

Formalin-fixed and paraffin-embedded samples (FFPE) tissues were obtained from primary biopsies at the Biomedical Research Institute INCLIVA (Valencia, Spain) and were subsequently treated following standard guidelines. All samples were analyzed by an expert pathologist to ensure tumor infiltration > 30%, and hormone receptors and HER2 status were evaluated by immunohistochemistry (IHC) and/or fluorescence in situ hybridization (FISH) in the latter case. MiRNAs-449 and *ACSL4* expression were analyzed by RT-qPCR in a discovery cohort of TNBC (*n* = 55 for miRNAs-449 and *n* = 33 for ACSL4) *versus* healthy tissues (*n* = 19) before treatment. Clinicopathological characteristics are described in Table S1 and S2, respectively. *ACSL4* expression was also analyzed in primary biopsies of non-relapsed (*n* = 12) and relapsed (*n* = 20) patients after receiving anthracyclines (neo)adjuvant-containing chemotherapy treatment. Clinical data acquisition was carried out in a period between 21 and 105 months. Clinicopathological characteristics are described in Table S3. Briefly, total RNA was isolated from FFPE tissue using the RecoverAll Total Nucleic Acid Kit (Ambion). Expression of housekeeping *GAPDH* mRNA or *miR-16* miRNA was used as an endogenous control. The study was approved on the 25th of June of 2015 by the Research Ethics Committee of the Hospital Clínico (ethical approval number 2014/178) and all patients signed the written informed consent.

### Statistical analyses

All statistical analyses were performed in GraphPad Prism version 8.0.1 software (La Jolla, USA). IC_50_ values were calculated using a variable slope (four parameters) curve. Mean comparisons were performed using the two-tailed Student’s *T*-test for normal distribution, otherwise the Mann–Whitney *U*-test was used. Receiver-operating characteristic (ROC) curves were performed by plotting sensitivity (true positive) *versus* 100-specificity (false positive), and the area under the curve (AUC) was calculated. The sensitivity and specificity were also calculated based on the highest value using Youden’s J index. Principal Component analysis (PCA) was used to summarize the miRNAs signature into a single score vector. This linear combination of all miRNAs is a weighted average, where each miRNA is weighted by its importance within the first principal component. A value of *p* < 0.05 was defined as statistically significant. Assays were performed in technical and biological triplicate.

### Supplementary information

Supplementary material 1

Supplementary material 2

## Data Availability

The datasets analyzed during the current study are available in the Kaplan–Meier plotter (https://kmplot.com/analysis/) and DIANA TOOLS- mirPath v.3 (http://www.microrna.gr/miRPathv3) repositories. The other data generated or analyzed during this study are included in this published article and its supplementary information files.

## References

[1] Sung H, Ferlay J, Siegel RL, Laversanne M, Soerjomataram I, Jemal A, et al. Global Cancer Statistics 2020: GLOBOCAN estimates of incidence and mortality worldwide for 36 cancers in 185 countries. CA Cancer J Clin. 2021;71:209–49. 33538338 10.3322/caac.21660

[2] Waks AG, Winer EP. Breast cancer treatment: a review. JAMA. 2019;321:288–300. 30667505 10.1001/jama.2018.19323

[3] O’Reilly D, Sendi MA, Kelly CM. Overview of recent advances in metastatic triple negative breast cancer. World J Clin Oncol. 2021;12:164–82. 33767972 10.5306/wjco.v12.i3.164 PMC7968109

[4] Yadav BS, Chanana P, Jhamb S. Biomarkers in triple negative breast cancer: a review. World J Clin Oncol. 2015;6:252–63. 26677438 10.5306/wjco.v6.i6.252 PMC4675910

[5] Kim C, Gao R, Sei E, Brandt R, Hartman J, Hatschek T, et al. Chemoresistance evolution in triple-negative breast cancer delineated by single cell sequencing. Cell. 2018;173:879–93. 29681456 10.1016/j.cell.2018.03.041 PMC6132060

[6] Christowitz C, Davis T, Isaacs A, Van Niekerk G, Hattingh S, Engelbrecht AM. Mechanisms of doxorubicin-induced drug resistance and drug resistant tumour growth in a murine breast tumour model. BMC Cancer. 2019;19:757. 31370818 10.1186/s12885-019-5939-z PMC6670209

[7] Shim GS, Manandhar S, Shin DH, Kim TH, Kwak MK. Acquisition of doxorubicin resistance in ovarian carcinoma cells accompanies activation of the NRF2 pathway. Free Radic Biol Med. 2009;47:1619–31. 19751820 10.1016/j.freeradbiomed.2009.09.006

[8] Xu F, Wang F, Yang T, Sheng Y, Zhong T, Chen Y. Differential drug resistance acquisition to doxorubicin and paclitaxel in breast cancer cells. Cancer Cell Int. 2014;14:142. 25550688 10.1186/s12935-014-0142-4 PMC4279688

[9] McGuirk S, Audet-Delage Y, Annis MG, Xue Y, Vernier M, Zhao K, et al. Resistance to different anthracycline chemotherapeutics elicits distinct and actionable primary metabolic dependencies in breast cancer. Elife. 2021;10:e65150. 34181531 10.7554/eLife.65150 PMC8238502

[10] Yun UJ, Lee JH, Shim J, Yoon K, Goh SH, Yi EH, et al. Anti-cancer effect of doxorubicin is mediated by downregulation of HMG-Co A reductase via inhibition of EGFR/Src pathway. Lab Investig. 2019;99:1157–72. 30700846 10.1038/s41374-019-0193-1

[11] Martin SL, Kala R, Tollefsbol TO. Mechanisms for inhibition of colon cancer cells by sulforaphane through epigenetic modulation and hTERT down-regulation. Curr Cancer Drug Targets. 2018;18:97–106. 28176652 10.2174/1568009617666170206104032 PMC5577390

[12] Lewis KA, Jordan HR, Tollefsbol TO. Effects of SAHA and EGCG on growth potentiation of triple-negative breast cancer cells. Cancers (Basel). 2019;11:23. 10.3390/cancers11010023 PMC6356328 30591655

[13] Li J, Song Y, Wang Y, Luo J, Yu W. MicroRNA-148a suppresses epithelial-to-mesenchymal transition by targeting ROCK1 in non-small cell lung cancer cells. Mol Cell Biochem. 2013;380:277–82. 23670799 10.1007/s11010-013-1682-y

[14] Yamada N, Noguchi S, Mori T, Naoe T, Maruo K, Akao Y. Tumor-suppressive microRNA-145 targets catenin δ-1 to regulate Wnt/β-catenin signaling in human colon cancer cells. Cancer Lett. 2013;335:332–42. 23499891 10.1016/j.canlet.2013.02.060

[15] Ying X, Wei K, Lin Z, Cui Y, Ding J, Chen Y, et al. MicroRNA-125b suppresses ovarian cancer progression via suppression of the epithelial-mesenchymal transition pathway by targeting the SET protein. Cellular Physiology and Biochemistry. 2016;39:501–10. 27383536 10.1159/000445642

[16] Bhaumik D, Scott GK, Schokrpur S, Patil CK, Campisi J, Benz C. Expression of microRNA-146 suppresses NF-κB activity with reduction of metastatic potential in breast cancer cells. oncoogene. 2008;27:5643–7. 10.1038/onc.2008.171 PMC2811234 18504431

[17] Jin M, Yang Z, Ye W, Xu H, Hua X. MicroRNA-150 predicts a favorable prognosis in patients with epithelial ovarian cancer, and inhibits cell invasion and metastasis by suppressing transcriptional repressor ZEB1. PLoS ONE. 2014;9:e103965. 25090005 10.1371/journal.pone.0103965 PMC4121232

[18] Saliminejad K, Khorram Khorshid HR, Soleymani Fard S, Ghaffari SH. An overview of microRNAs: Biology, functions, therapeutics, and analysis methods. J Cell Physiol. 2019;234:5451–65. 30471116 10.1002/jcp.27486

[19] Yong-Ming H, Ai-Jun J, Xiao-Yue X, Jian-Wei L, Chen Y, Ye C. MiR-449a: a potential therapeutic agent for cancer. Anticancer Drugs. 2017;28:1067–78. 29023247 10.1097/CAD.0000000000000555

[20] Lizé M, Pilarski S, Dobbelstein M. E2F1-inducible microRNA 449a/b suppresses cell proliferation and promotes apoptosis. Cell Death Differ. 2010;17:452–8. 19960022 10.1038/cdd.2009.188

[21] Chen SP, Liu BX, Xu J, Pei XF, Liao YJ, Yuan F, et al. MiR-449a suppresses the epithelial-mesenchymal transition and metastasis of hepatocellular carcinoma by multiple targets. BMC Cancer. 2015;15:706. 26471185 10.1186/s12885-015-1738-3 PMC4608176

[22] Yang X, Feng M, Jiang X, Wu Z, Li Z, Aau M, et al. miR-449a and miR-449b are direct transcriptional targets of E2F1 and negatively regulate pRb–E2F1 activity through a feedback loop by targeting CDK6 and CDC25A. Genes Dev. 2009;23:2388–93. 19833767 10.1101/gad.1819009 PMC2764491

[23] Hou Y, Feng F, Yang R. Effect of miR-449a-mediated Notch signaling pathway on the proliferation, apoptosis and invasion of papillary thyroid carcinoma cells. Oncol Rep. 2020;43:471–80. 31894345 10.3892/or.2019.7443 PMC6967094

[24] Luo W, Huang B, Li Z, Li H, Sun L, Zhang Q, et al. MicroRNA-449a is downregulated in non-small cell lung cancer and inhibits migration and invasion by targeting c-Met. PLoS ONE. 2013;8:e64759. 23734217 10.1371/journal.pone.0064759 PMC3667122

[25] Noonan EJ, Place RF, Pookot D, Basak S, Whitson JM, Hirata H, et al. miR-449a targets HDAC-1 and induces growth arrest in prostate cancer. Oncogene. 2009;28:1714–24. 19252524 10.1038/onc.2009.19

[26] Sun X, Liu S, Chen P, Fu D, Hou Y, Hu J. miR-449a inhibits colorectal cancer progression by targeting SATB2. Oncotarget. 2017;8:100975–88. 29254139 10.18632/oncotarget.10900 PMC5731849

[27] Buurman R, Grlevik E, Schffer V, Eilers M, Sandbothe M, Kreipe H, et al. Histone deacetylases activate hepatocyte growth factor signaling by repressing microRNA-449 in hepatocellular carcinoma cells. Gastroenterology. 2012;143:811–820.e15. 22641068 10.1053/j.gastro.2012.05.033

[28] Sandbothe M, Buurman R, Reich N, Greiwe L, Vajen B, Gürlevik E, et al. The microRNA-449 family inhibits TGF-β-mediated liver cancer cell migration by targeting SOX4. J Hepatol. 2017;66:1012–21. 28088579 10.1016/j.jhep.2017.01.004

[29] Ma Y, Zhang X, Alsaidan OA, Yang X, Sulejmani E, Zha J, et al. Long-chain acyl-CoA synthetase 4–mediated fatty acid metabolism sustains androgen receptor pathway–independent prostate cancer. Mol Cancer Res. 2021;19:124–35. 33077484 10.1158/1541-7786.MCR-20-0379 PMC7785683

[30] Dattilo MA, Benzo Y, Herrera LM, Prada JG, Castillo AF, Orlando UD, et al. Regulatory mechanisms leading to differential Acyl-CoA synthetase 4 expression in breast cancer cells. Sci Rep. 2019;9:10324. 31311992 10.1038/s41598-019-46776-7 PMC6635356

[31] Wu X, Li Y, Wang J, Wen X, Marcus MT, Daniels G, et al. Long chain fatty acyl-CoA synthetase 4 is a biomarker for and mediator of hormone resistance in human breast cancer. PLoS ONE. 2013;8:e77060. 24155918 10.1371/journal.pone.0077060 PMC3796543

[32] Tormo E, Ballester S, Adam-Artigues A, Burgués O, Alonso E, Bermejo B, et al. The miRNA-449 family mediates doxorubicin resistance in triple-negative breast cancer by regulating cell cycle factors. Sci Rep. 2019;9:5316. 30926829 10.1038/s41598-019-41472-y PMC6441107

[33] Zhang L, Huang J, Yang N, Greshock J, Megraw MS, Giannakakis A, et al. microRNAs exhibit high frequency genomic alterations in human cancer. Proc Natl Acad Sci USA. 2006;103:9136–41. 16754881 10.1073/pnas.0508889103 PMC1474008

[34] Moore LD, Le T, Fan G. DNA methylation and its basic function. Neuropsychopharmacology. 2013;38:23–38. 22781841 10.1038/npp.2012.112 PMC3521964

[35] Gabay M, Li Y, Felsher DW. MYC activation is a hallmark of cancer initiation and maintenance meital. Cold Spring Harb Perspect Med. 2009;36:186–94. 10.1101/cshperspect.a014241 PMC4031954 24890832

[36] Torrezan GT, Ferreira EN, Nakahata AM, Barros BDF, Castro MTM, Correa BR, et al. Recurrent somatic mutation in DROSHA induces microRNA profile changes in Wilms tumour. Nat Commun. 2014;5:4039. 24909261 10.1038/ncomms5039 PMC4062040

[37] Peng Y, Croce CM. The role of microRNAs in human cancer. Signal Transduct Target Ther. 2016;1:15004. 29263891 10.1038/sigtrans.2015.4 PMC5661652

[38] Jiang J, Yang X, He X, Ma W, Wang J, Zhou Q, et al. MicroRNA-449b-5p suppresses the growth and invasion of breast cancer cells via inhibiting CREPT-mediated Wnt/β-catenin signaling. Chem Biol Interact. 2019;302:74–82. 30738779 10.1016/j.cbi.2019.02.004

[39] Shi W, Bruce J, Lee M, Yue S, Rowe M, Pintilie M, et al. MiR-449a promotes breast cancer progression by targeting CRIP2. Oncotarget. 2016;7:18906–18. 26934316 10.18632/oncotarget.7753 PMC4951339

[40] Zhang Z, Wang J, Gao R, Yang X, Zhang Y, Li J, et al. Downregulation of MicroRNA-449 promotes migration and invasion of breast cancer cells by targeting tumor protein D52 (TPD52). Oncol Res. 2017;25:753–61. 27983918 10.3727/096504016X14772342320617 PMC7841004

[41] Wei KL, Cao XM, Xiong DD, Zeng JJ, Lan AH, Chen G, et al. Downregulation of miRNA-449a expression associated with advanced stages and lymph node metastasis of breast cancer. Int J Clin Exp Pathol. 2016;9:7370–80.

[42] Ishikawa D, Takasu C, Kashihara H, Nishi M, Tokunaga T, Higashijima J, et al. The significance of microRNA-449a and its potential target HDAC1 in patients with colorectal cancer. Anticancer Res. 2019;39:2855–60. 31177123 10.21873/anticanres.13414

[43] Wang C, Chen L, Hou X, Li Z, Kabra N, Ma Y, et al. Interactions between E2F1 and SirT1 regulate apoptotic response to DNA damage. Nat Cell Biol. 2006;8:1025–31. 16892051 10.1038/ncb1468

[44] Robertson KD, Ait-Si-Ali S, Yokochi T, Wade PA, Jones PL, Wolffe AP. DNMT1 forms a complex with RB, E2F1 and HDAC1 and represses transcription from E2F-responsive promoters. Nat Genet. 2000;25:338–42. 10888886 10.1038/77124

[45] Zhang X, Liu H, Xie Z, Deng W, Wu C, Qin B, et al. Epigenetically regulated miR-449a enhances hepatitis B virus replication by targeting cAMP-responsive element binding protein 5 and modulating hepatocytes phenotype. Sci Rep. 2016;6:25389. 27138288 10.1038/srep25389 PMC4853741

[46] Poddar S, Kesharwani D, Datta M. Histone deacetylase inhibition regulates miR-449a levels in skeletal muscle cells. Epigenetics. 2016;11:579–87. 27184529 10.1080/15592294.2016.1188247 PMC4990227

[47] Kheir TB, Futoma-kazmierczak E, Jacobsen A, Krogh A, Bardram L, Hother C, et al. miR-449 inhibits cell proliferation and is down-regulated in gastric cancer. Mol Cancer. 2011;10:29. 21418558 10.1186/1476-4598-10-29 PMC3070685

[48] Germain N, Dhayer M, Boileau M, Fovez Q, Kluza J, Marchetti P. Lipid metabolism and resistance to anticancer treatment. Biology. 2020;9:474. 33339398 10.3390/biology9120474 PMC7766644

[49] Radif Y, Ndiaye H, Kalantzi V, Jacobs R, Hall A, Minogue S, et al. The endogenous subcellular localisations of the long chain fatty acid-activating enzymes ACSL3 and ACSL4 in sarcoma and breast cancer cells. Mol Cell Biochem. 2018;448:275–86. 29450800 10.1007/s11010-018-3332-x PMC6182735

[50] Yoon S, Lee MY, Park SW, Moon JS, Koh YK, Ahn YH, et al. Up-regulation of acetyl-CoA carboxylase α and fatty acid synthase by human epidermal growth factor receptor 2 at the translational level in breast cancer cells. J Biol Chem. 2007;282:26122–31. 17631500 10.1074/jbc.M702854200

[51] Ferraro GB, Ali A, Luengo A, Kodack DP, Deik A, Abbott KL, et al. Fatty acid synthesis is required for breast cancer brain metastasis. Nat Cancer. 2021;2:414–28. 34179825 10.1038/s43018-021-00183-y PMC8223728

[52] Menendez JA, Lupu R. Fatty acid synthase (FASN) as a therapeutic target in breast cancer. Expert Opin Ther Targets. 2017;21:1001–16. 28922023 10.1080/14728222.2017.1381087

[53] Monaco ME. Fatty acid metabolism in breast cancer subtypes. Oncotarget. 2017;8:29487–500. 28412757 10.18632/oncotarget.15494 PMC5438746

[54] Maloberti PM, Duarte AB, Orlando UD, Pasqualini ME, Solano AR, Lopez-Otín C, et al. Functional interaction between acyl-coa synthetase 4, lipooxygenases and cyclooxygenase-2 in the aggressive phenotype of breast cancer cells. PLoS ONE. 2010;5:e15540. 21085606 10.1371/journal.pone.0015540 PMC2978721

[55] Monaco ME, Creighton CJ, Lee P, Zou X, Topham MK, Stafforini DM. Expression of long-chain fatty acyl-CoA synthetase 4 in breast and prostate cancers is associated with sex steroid hormone receptor negativity. Transl Oncol. 2010;3:91–8. 20360933 10.1593/tlo.09202 PMC2847316

[56] Orlando UD, Castillo AF, Dattilo MA, Solano AR, Maloberti PM, Podesta EJ. Acyl-CoA synthetase-4, a new regulator of mTOR and a potential therapeutic target for enhanced estrogen receptor function in receptor-positive and -negative breast cancer. Oncotarget. 2015;6:42632–50. 26536660 10.18632/oncotarget.5822 PMC4767459

[57] Yun MR, Lim SM, Kim SK, Choi HM, Pyo KH, Kim SK, et al. Enhancer remodeling and microRNA alterations are associated with acquired resistance to ALK inhibitors. Cancer Res. 2018;78:3350–62. 29669761 10.1158/0008-5472.CAN-17-3146

[58] Li Q, Li H, Zhao X, Wang B, Zhang L, Zhang C, et al. DNA methylation mediated downregulation of miR-449c controls osteosarcoma cell cycle progression by directly targeting oncogene c-Myc. Int J Biol Sci. 2017;13:1038–50. 28924385 10.7150/ijbs.19476 PMC5599909

[59] Chikh A, Ferro R, Abbott JJ, Piñeiro R, Buus R, Iezzi M, et al. Class II phosphoinositide 3-kinase C2β regulates a novel signaling pathway involved in breast cancer progression. Oncotarget. 2016;7:18325–45. 26934321 10.18632/oncotarget.7761 PMC4951291

[60] Mao A, Zhao Q, Zhou X, Sun C, Si J, Zhou R, et al. MicroRNA-449a enhances radiosensitivity by downregulation of c-Myc in prostate cancer cells. Sci Rep. 2016;6:27346. 27250340 10.1038/srep27346 PMC4890029

[61] Mao A, Liu Y, Wang Y, Zhao Q, Zhou X, Sun C, et al. miR-449a enhances radiosensitivity through modulating pRb/E2F1 in prostate cancer cells. Tumor Biology. 2016;37:4831–40. 26520443 10.1007/s13277-015-4336-8

[62] Li L, Liu H, Du L, Xi P, Wang Q, Li Y, et al. MiR-449a suppresses LDHA-mediated glycolysis to enhance the sensitivity of non-small cell lung cancer cells to ionizing radiation. Oncol Res. 2018;26:547–56. 28800787 10.3727/096504017X15016337254605 PMC7844793

[63] Zhou Y, Chen Q, Qin R, Zhang K, Li H. MicroRNA-449a reduces cell survival and enhances cisplatin-induced cytotoxicity via downregulation of NOTCH1 in ovarian cancer cells. Tumor Biology. 2014;35:12369–78. 25179844 10.1007/s13277-014-2551-3

[64] Vajen B, Bhowmick R, Greiwe L, Schäffer V, Eilers M, Reinkens T, et al. MicroRNA-449a inhibits triple negative breast cancer by disturbing DNA repair and chromatid separation. Int J Mol Sci. 2022;23:5131. 35563522 10.3390/ijms23095131 PMC9102308

[65] Castillo AF, Orlando UD, Maloberti PM, Prada JG, Dattilo MA, Solano AR, et al. New inhibitor targeting Acyl-CoA synthetase 4 reduces breast and prostate tumor growth, therapeutic resistance and steroidogenesis. Cell Mol Life Sci. 2021;78:2893–910. 33068124 10.1007/s00018-020-03679-5 PMC11072814

[66] Jiang X, Guo S, Zhang Y, Zhao Y, Li X, Jia Y, et al. LncRNA NEAT1 promotes docetaxel resistance in prostate cancer by regulating ACSL4 via sponging miR-34a-5p and miR-204-5p. Cell Signal. 2020;65:109422. 31672604 10.1016/j.cellsig.2019.109422

[67] Orlando UD, Castillo AF, Medrano MAR, Solano AR, Maloberti PM, Podesta EJ. Acyl-CoA synthetase-4 is implicated in drug resistance in breast cancer cell lines involving the regulation of energy-dependent transporter expression. Biochem Pharmacol. 2019;159:52–63. 30414939 10.1016/j.bcp.2018.11.005

[68] Chen J, Ding C, Chen Y, Hu W, Lu Y, Wu W, et al. ACSL4 promotes hepatocellular carcinoma progression via c-Myc stability mediated by ERK/FBW7/c-Myc axis. Oncogenesis. 2020;9:42. 32350243 10.1038/s41389-020-0226-z PMC7190855

[69] Zhang Y, Li S, Li F, Lv C, Yang QK. High-fat diet impairs ferroptosis and promotes cancer invasiveness via downregulating tumor suppressor ACSL4 in lung adenocarcinoma. Biol Direct. 2021;16:10. 34053456 10.1186/s13062-021-00294-7 PMC8166005

[70] Lv Y, Feng QY, Zhang ZY, Zheng P, Zhu DX, Lin Q, et al. Low ferroptosis score predicts chemotherapy responsiveness and immune-activation in colorectal cancer. Cancer Med. 2022;12:2033–45. 10.1002/cam4.4956 PMC9883409 35855531

[71] Yu Y, Sun X, Chen F, Liu M. Genetic alteration, prognostic and immunological role of acyl-CoA synthetase long-chain family member 4 in a pan-cancer analysis. Front Genet. 2022;13:812674. 35126480 10.3389/fgene.2022.812674 PMC8811308

[72] Guo N. Identification of ACSL4 as a biomarker and contributor of ferroptosis in clear cell renal cell carcinoma. Transl Cancer Res. 2022;11:2688–99. 36093519 10.21037/tcr-21-2157 PMC9459583

[73] Xiao H, Zheng Y, Ma L, Tian L, Sun Q. Clinically-relevant ABC transporter for anti-cancer drug resistance. Front Pharmacol. 2021;12:648407. 33953682 10.3389/fphar.2021.648407 PMC8089384

[74] Shu H, Yuan B, Huang Y, Wang L, He B, Sun Q, et al. High expression of ABCG2 is associated with chemotherapy resistance of osteosarcoma. J Orthop Surg Res. 2021;16:85. 33509236 10.1186/s13018-021-02204-z PMC7842061

[75] Kukal S, Guin D, Rawat C, Bora S, Mishra MK, Sharma P, et al. Multidrug efflux transporter ABCG2: expression and regulation. Cell Mol Life Sci. 2021;78:6887–939. 34586444 10.1007/s00018-021-03901-y PMC11072723

[76] Lánczky A, Győrffy B. Web-based survival analysis tool tailored for medical research (KMplot): development and implementation. J Med Internet Res. 2021;23:e27633. 34309564 10.2196/27633 PMC8367126

