# Supplementary material for: MiRNA-449 family is epigenetically repressed and sensitizes to doxorubicin through ACSL4 downregulation in triple-negative breast cancer
Source: Cell Death Discov. 2024 Aug 22;10:372. doi: 10.1038/s41420-024-02128-7 (PMC11341569; doi:10.1038/s41420-024-02128-7)
Supplement: Supplementary file 1 — Supplementary material 1 [file 41420_2024_2128_MOESM1_ESM.docx]

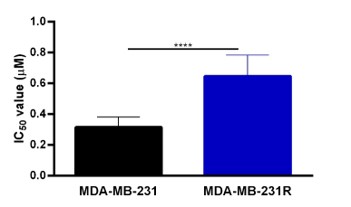


**Fig. S1**: **MDA-MB-231R** **presents doxorubicin-acquired resistance.** The IC_50_ values were determined by WST-1 after treatment with different concentrations of doxorubicin for 48 hours in MDA-MB-231 (0.32 ± 0.07 µM) and MDA-MB-231R (0.65 ± 0.14 µM) cell lines (mean ±. SD) (*n* = 3) **** *p* < 0.0001.


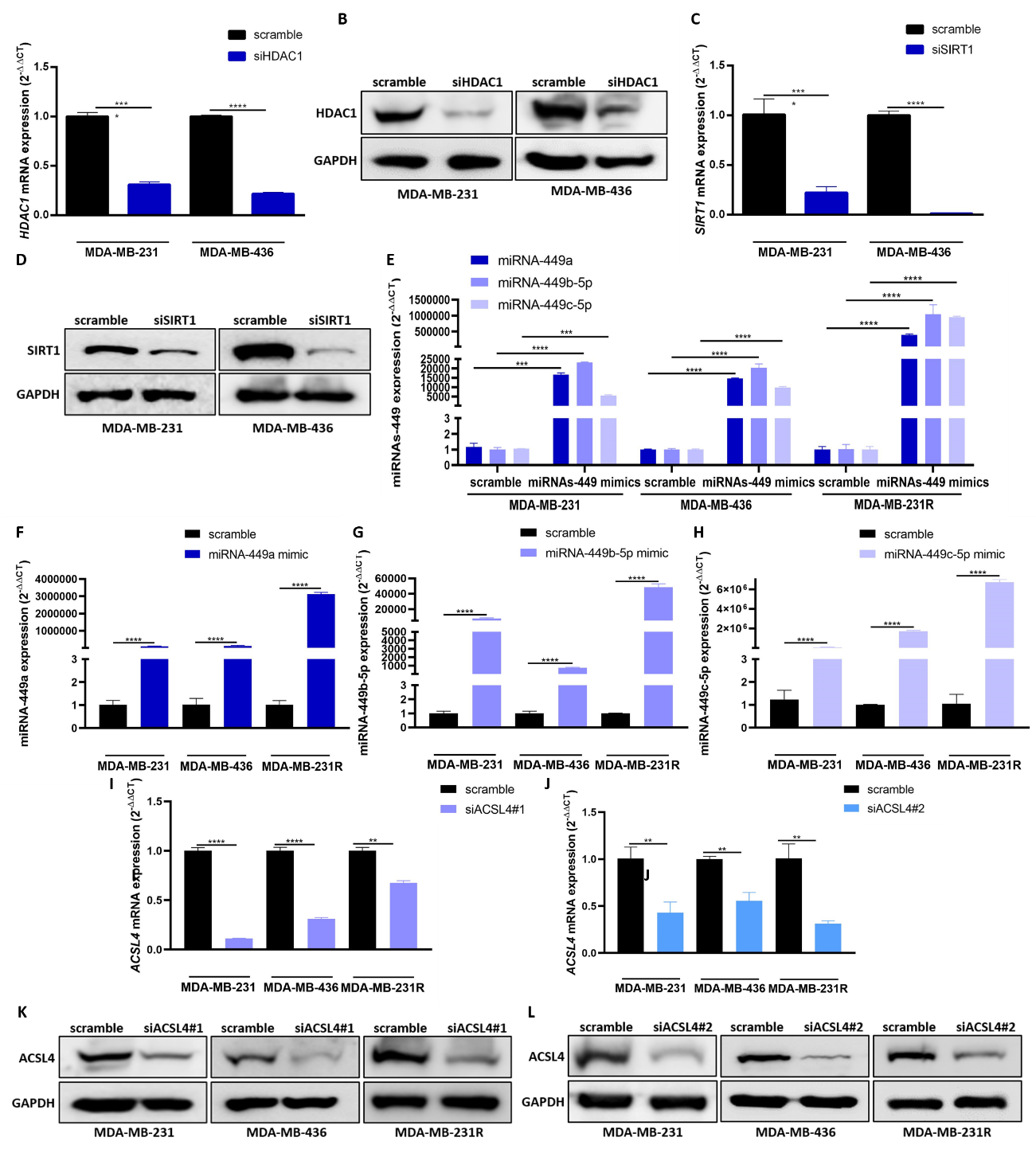


**Fig. S2: Confirmation of cell transfection in TNBC cell lines.** (**A-B**) HDAC1 expression was analyzed by RT-qPCR (**A**) (mean ±. SD) (*n* = 3) and Western blot (**B**) in MDA-MB-231 and MDA-MB-436 cell lines after siHDAC1 transfection. (**C-D**) SIRT1 expression was analyzed by RT-qPCR (**c**) (mean ±. SD) (*n* = 3) and Western blot (**D**) in MDA-MB-231 and MDA-MB-436 cell lines after siSIRT1 transfection. (**E**) MiRNAs-449 expression was analyzed by RT-qPCR in MDA-MB-231, MDA-MB-436, and MDA-MB-231R cell lines after miRNAs-449 mimic transfection (mean ±. SD) (*n* = 3). (**F**) miRNA-449a, (**G**) miRNA-449b-5p and (**H**) miRNA-449c-5p expression were analyzed by RT-qPCR in MDA-MB-231, MDA-MB-436 and MDA-MB-231R cell lines after miRNA-449a, miRNA-449b-4p and miRNA-449c-5p mimics transfection separately, respectively (mean ±. SD) (*n* = 3). (**I-L**) ACSL4 expression was analyzed by RT-qPCR (**I, J**) (mean ±. SD) (*n* = 3) and Western blot (**K, L**) in MDA-MB-231, MDA-MB-436, and MDA-MB-231R cell lines after two siRNAs targeting *ACSL4* (siACSL4#1 and siACSL4#2). ***p* < 0.01, ****p* < 0.001, *****p* < 0.0001.


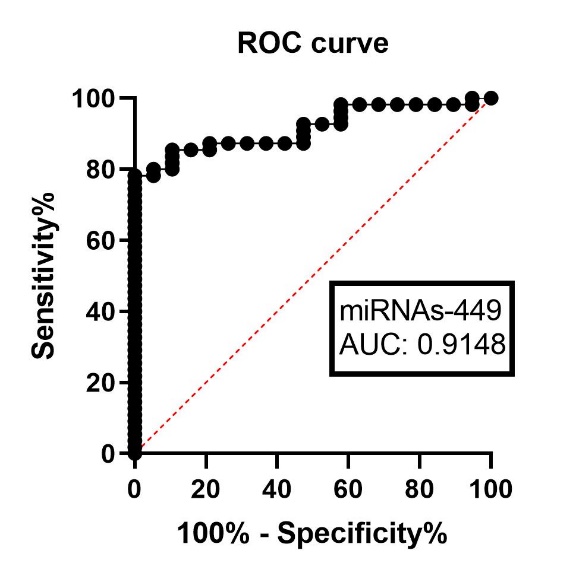


**Fig. S3:** **The signature of miRNAs-449 as a diagnostic biomarker.** ROC curve analyses were performed for miRNAs-449 signature in TNBC tissue samples (*n* = 55) and healthy tissue samples (*n* = 23).


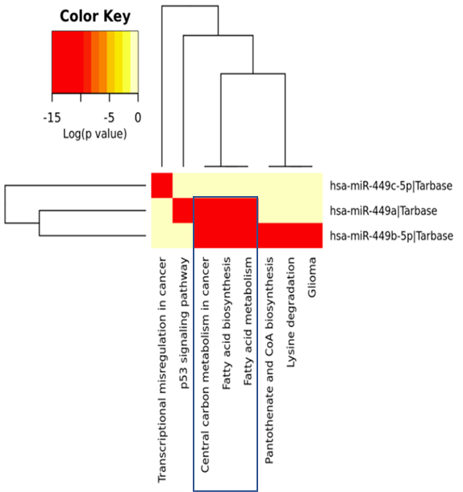


**Fig. S4:** **MiRNAs-449 modulate fatty acid metabolism.** MiRpath software analysis of miRNAs-449 targeted pathways. The color indicates a logarithmic transformation of Fisher’s Exact test-adjusted p-values (red for lower p-values).


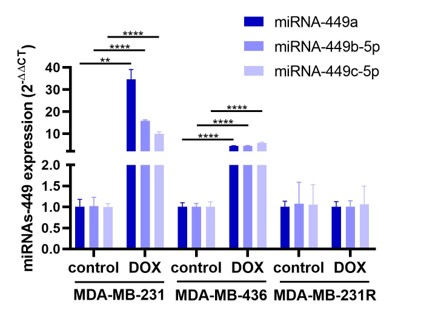


**Fig. S5: MiRNAs-449 are upregulated after doxorubicin treatment in doxorubicin-sensitive but not in doxorubicin-resistant cells.** MiRNA-449 family (miRNA-449a, miRNA-449b-5p, and miRNA-449c-5p) expression was analyzed by RT-qPCR in MDA-MB-231, MDA-MB-436, and MDA-MB-231R cell lines after doxorubicin treatment (1 µM, 48 hours) (mean ±. SD) (*n* = 3). ***p* < 0.01, *****p* < 0.0001.

**
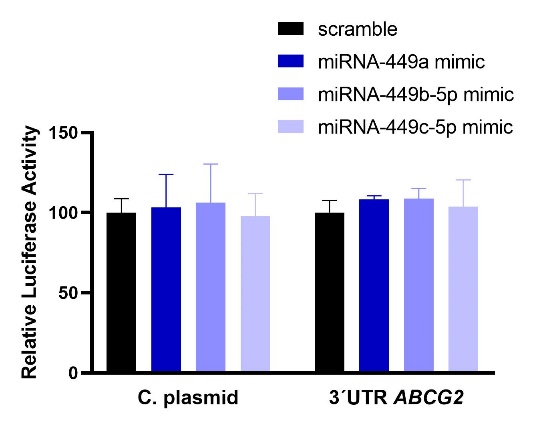
**

**Fig. S6: *ABCG2* is not a direct target of miRNAs-449.** Luciferase reporter assay was performed in HEK-293T cell line co-transfected with pEZX-MT06 (3’UTR ABCG2 containing or empty vector) and miRNA-449a, miRNA-449b-5p or miRNA-449c-5p mimics separately (mean ± SD) (*n* = 4). C plasmid: Control plasmid.

**Table S1: Clinicopathological characteristics of TNBC patients and controls for miRNAs-449 analyses expression.**

| **Characteristics** | **TNBC patients** | **Control** |
| --- | --- | --- |
| **Number** | 55 | 19 |
| **Grade group, n (%)** |  |  |
| 1 | 0(0%) | n.a |
| 2 | 11(20%) |  |
| 3 | 41(74.55%) |  |
| Unknown | 3(5.45%) |  |
| **Clinical stage, n (%)** |  |  |
| 1 | 13(23.64%) | n.a |
| 2 | 33(60%) |  |
| 3 | 8(14.54%) |  |
| 4 | 0(0%) |  |
| Unknown | 1(1.82%) |  |
| **Treatment, n (%)** |  |  |
| Neoadjuvant | 37(67.27%) | n.a |
| Adjuvant | 0(0%) |  |
| Neoadjuvant and Adjuvant | 18(32.73%) |  |
| **RCB, n (%)** |  |  |
| 0 | 22(40%) | n.a |
| 1 | 5(9.1%) |  |
| 2 | 16(29.1%) |  |
| 3 | 6(10.9%) |  |
| Unknown | 6(10.9%) |  |
| **Ki67, n (%)** |  |  |
| <30 | 4(7.27%) | n.a |
| 30-59.9 | 15(27.27%) |  |
| ≥60 | 33(60%) |  |
| Unknown | 3(5.46%) |  |
| **Regional lymph node metastasis, n (%)** |  |  |
| Yes | 19(34.54%) | n.a |
| No | 35(63.64%) |  |
| Unknown | 1(1.82%) |  |
| **Distant metastasis, n (%)** |  |  |
| Yes | 0(0%) | n.a |
| No | 54(98.18%) |  |
| Unknown | 1(1.82%) |  |

TNBC: Triple-negative breast cancer; RCB: Residual Cancer Burden; n.a: not applicable

**Table S2: Clinicopathological characteristics of TNBC patients and controls for *ACSL4* analyses expression.**

| **Characteristics** | **TNBC patients** | **Control** |
| --- | --- | --- |
| **Number** | 33 | 19 |
| **Grade group, n (%)** |  |  |
| 1 | 0(0%) | n.a |
| 2 | 7(21.21%) |  |
| 3 | 25(75.76%) |  |
| Unknown | 1(3.03%) |  |
| **Clinical stage, n (%)** |  |  |
| 1 | 8(24.24%) | n.a |
| 2 | 20(60.61%) |  |
| 3 | 5(15.15%) |  |
| 4 | 0(0%) |  |
| Unknown | 0(0%) |  |
| **Treatment, n (%)** |  |  |
| Neoadjuvant | 21(63.63%) | n.a |
| Adjuvant | 0(0%) |  |
| Neoadjuvant and Adjuvant | 8(36.37%) |  |
| **RCB, n (%)** |  |  |
| 0 | 17(51.51%) | n.a |
| 1 | 2(6.06%) |  |
| 2 | 9(27.27%) |  |
| 3 | 3(9.1%) |  |
| Unknown | 2(6.06%) |  |
| **Ki67, n (%)** |  |  |
| <30 | 3(9.1%) | n.a |
| 30-59.9 | 7(21.21%) |  |
| ≥60 | 23(69.69%) |  |
| Unknown | 0(0%) |  |
| **Regional lymph node metastasis, n (%)** |  |  |
| Yes | 10(30.3%) | n.a |
| No | 22(66.67%) |  |
| Unknown | 1(3.03%) |  |
| **Distant metastasis, n (%)** |  |  |
| Yes | 0(0%) | n.a |
| No | 33(100%) |  |
| Unknown | 0(0%) |  |

TNBC: Triple-negative breast cancer; RCB: Residual Cancer Burden; n.a: not applicable.

**Table S3: Clinicopathological characteristics of relapse and non-relapse TNBC patients after chemotherapy treatment for *ACSL4* analysis expression.**

| **Characteristics** | **TNBC no relapse patients** | **TNBC relapse patients** |
| --- | --- | --- |
| **Number** | 12 | 20 |
| **Grade group, n (%)** |  |  |
| 1 | 0(0%) | 0(0%) |
| 2 | 4(33.33%) | 3(15%) |
| 3 | 7(58.34%) | 17(85%) |
| Unknown | 1(8.33%) | 0(0%) |
| **Clinical stage, n (%)** |  |  |
| 1 | 6(50%) | 2(10%) |
| 2 | 5(41.67%) | 14(70%) |
| 3 | 1(8.33%) | 4(20%) |
| 4 | 0(0%) | 0(0%) |
| Unknown | 0(0%) | 0(0%) |
| **Treatment, n (%)** |  |  |
| Neoadjuvant | 11(91.67%) | 14(70%) |
| Adjuvant | 0(0%) | 0(0%) |
| Neoadjuvant and Adjuvant | 1(8.33%) | 6(30%) |
| **RCB, n (%)** |  |  |
| 0 | 10(83.34%) | 7(35%) |
| 1 | 1(8.33%) | 1(5%) |
| 2 | 1(8.33%) | 9(45%) |
| 3 | 0(0%) | 2(10%) |
| Unknown | 0(0%) | 1(5%) |
| **Ki67, n (%)** |  |  |
| <30 | 1(8.33%) | 1(5%) |
| 30-59.9 | 3(25%) | 4(20%) |
| ≥60 | 7(58.34%) | 15(75%) |
| Unknown | 1(8.33%) | 0(0%) |
| **Regional lymph node metastasis, n (%)** |  |  |
| Yes | 3(25%) | 6(30%) |
| No | 9(75%) | 13(65%) |
| Unknown | 0(0%) | 1(5%) |
| **Distant metastasis, n (%)** |  |  |
| Yes | 0(0%) | 0(0%) |
| No | 12(100%) | 19(95%) |
| Unknown | 0(0%) | 1(5%) |

TNBC: Triple-negative breast cancer; RCB: Residual Cancer Burden; n.a: not applicable.
