# Supplementary material for: MiRNA-449 family is epigenetically repressed and sensitizes to doxorubicin through ACSL4 downregulation in triple-negative breast cancer
Source: Cell Death Discov. 2024 Aug 22;10:372. doi: 10.1038/s41420-024-02128-7 (PMC11341569; doi:10.1038/s41420-024-02128-7)
Supplement: Supplementary file 2 — Supplementary material 2 [file 41420_2024_2128_MOESM2_ESM.docx]

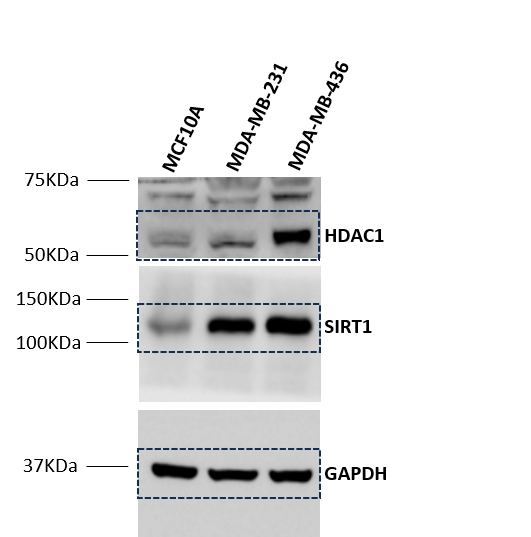


**Uncropped western blotting membranes of Figure 2C**. Abbreviations: HDAC1, histone deacetylase 1; SIRT1, sirtuin 1.


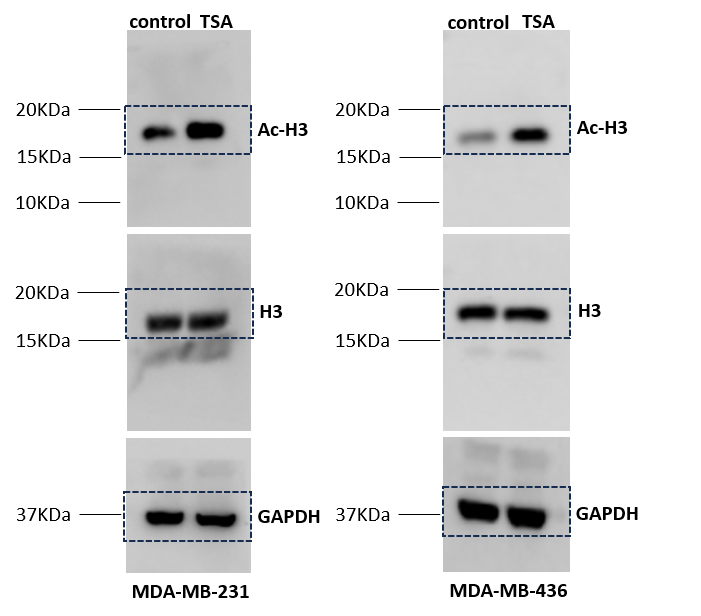


**Uncropped western blotting membranes of Figure 2F**. Abbreviations: Ac-H3, acetyl histone 3; H3, histone 3.


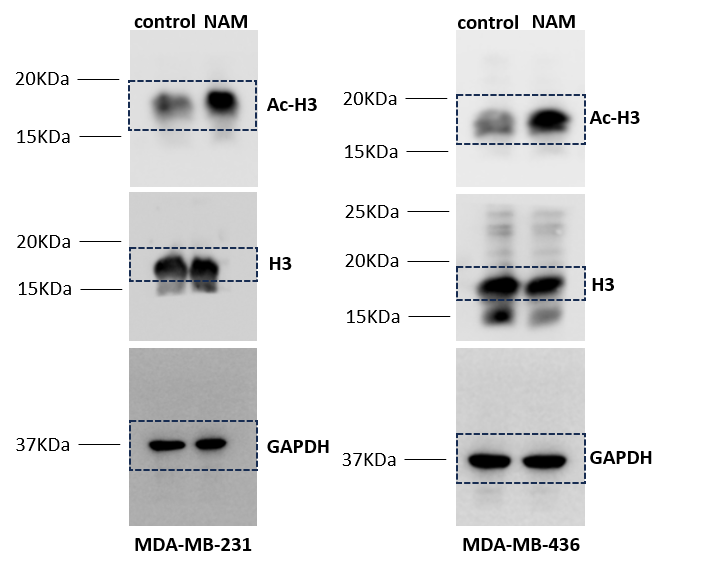


**Uncropped western blotting membranes of Figure 2G**. Abbreviations: Ac-H3, acetyl histone 3; H3, histone 3.


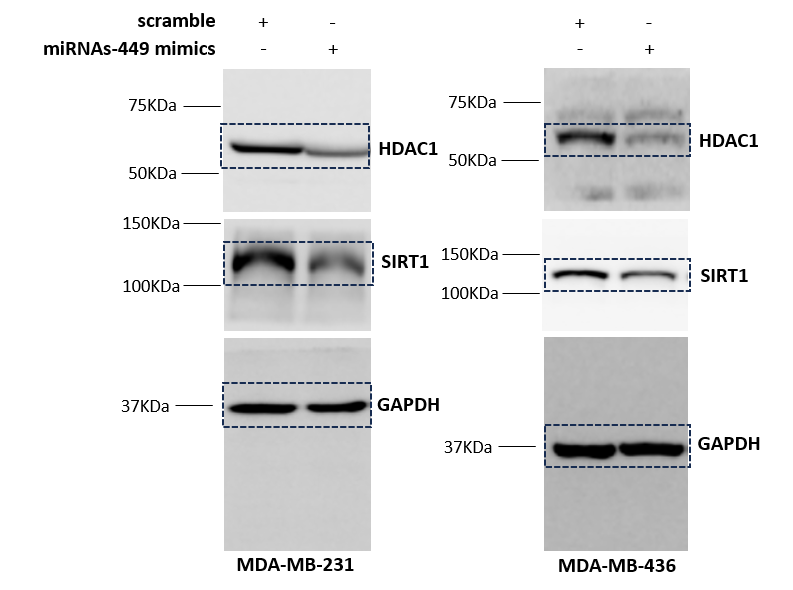


**Uncropped western blotting membranes of Figure 3C**. Abbreviations: HDAC1, histone deacetylase 1; SIRT1, sirtuin 1.


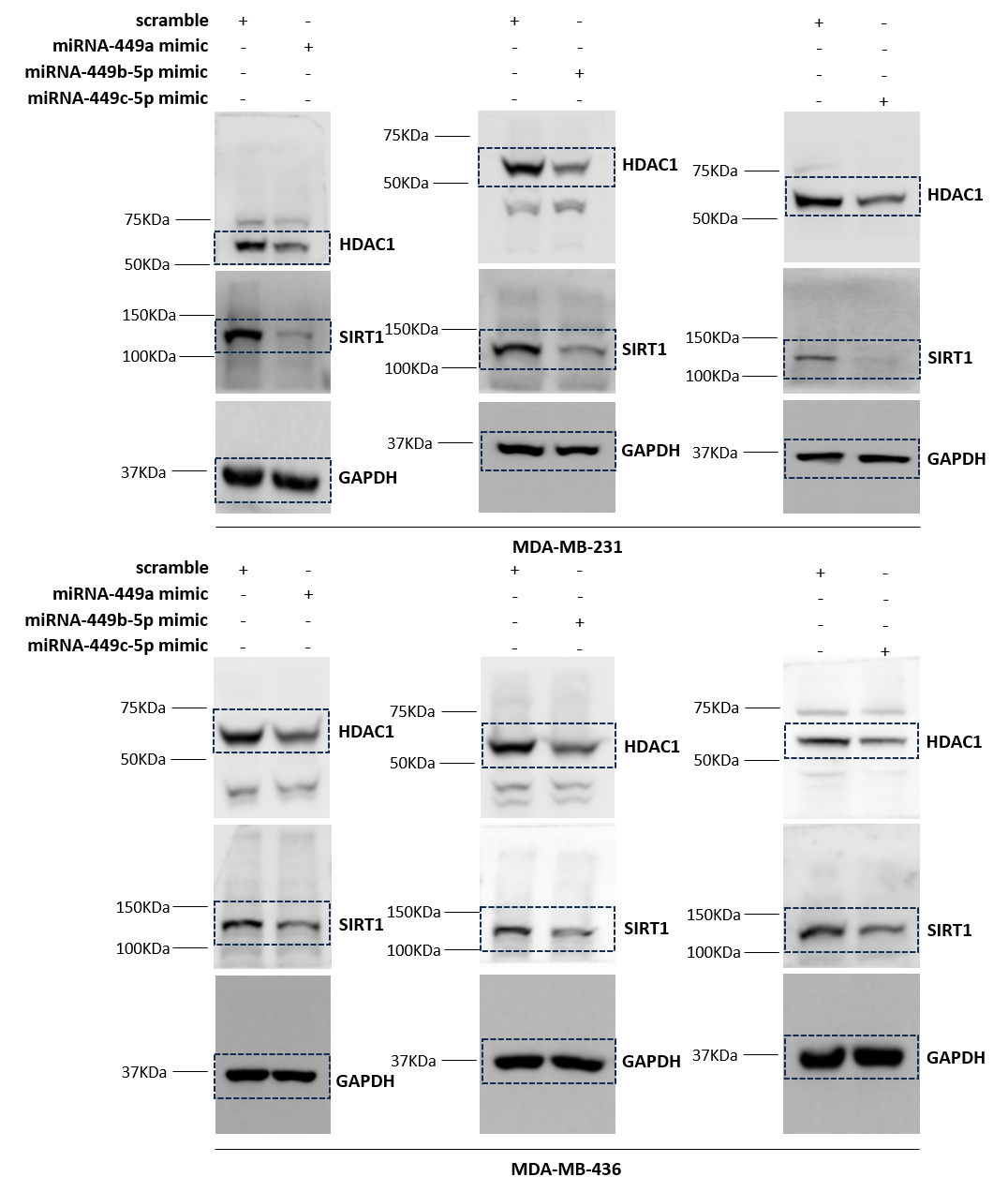


**Uncropped western blotting membranes of Figure 3F**. Abbreviations: HDAC1, histone deacetylase 1; SIRT1, sirtuin 1.


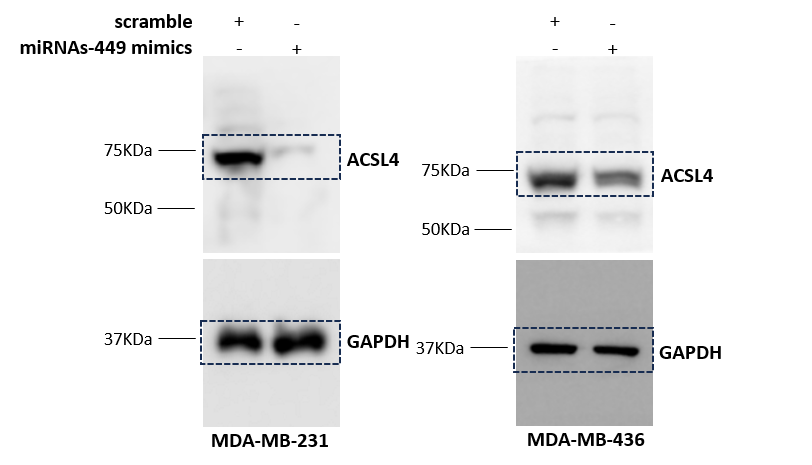


**Uncropped western blotting membranes of Figure 4D**. Abbreviations: ACSL4, acyl-CoA synthetase long-chain family member 4.


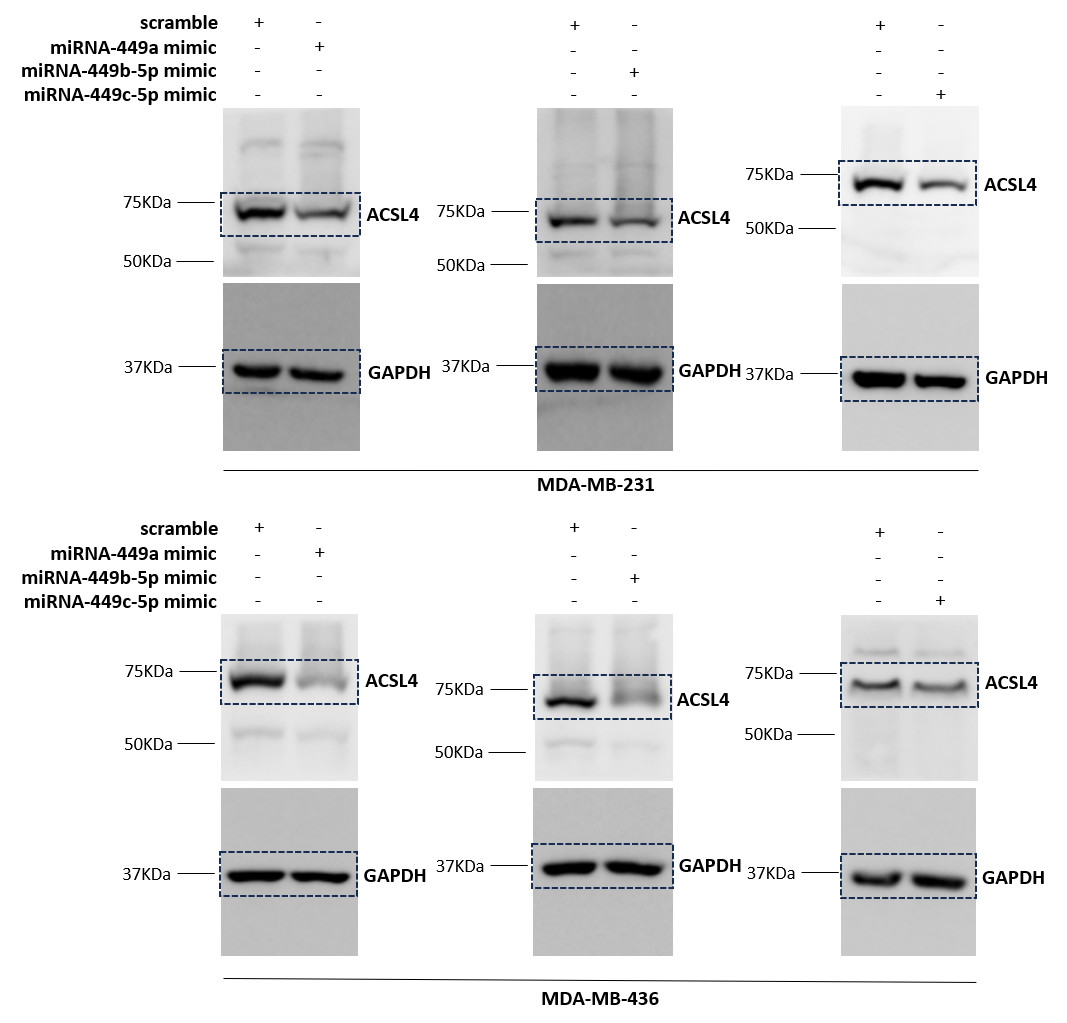


**Uncropped western blotting membranes of Figure 4F**. Abbreviations: ACSL4, acyl-CoA synthetase long-chain family member 4.


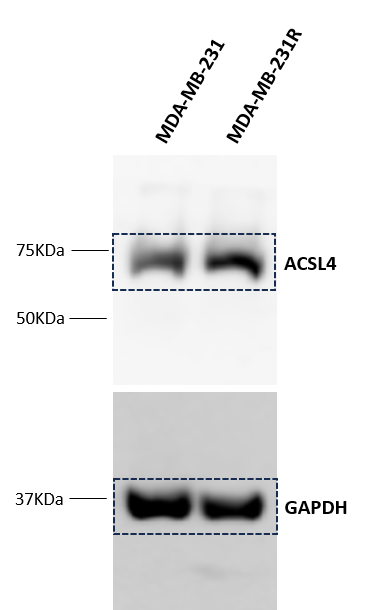


**Uncropped western blotting membranes of Figure 5C**. Abbreviations: ACSL4, acyl-CoA synthetase long-chain family member 4.


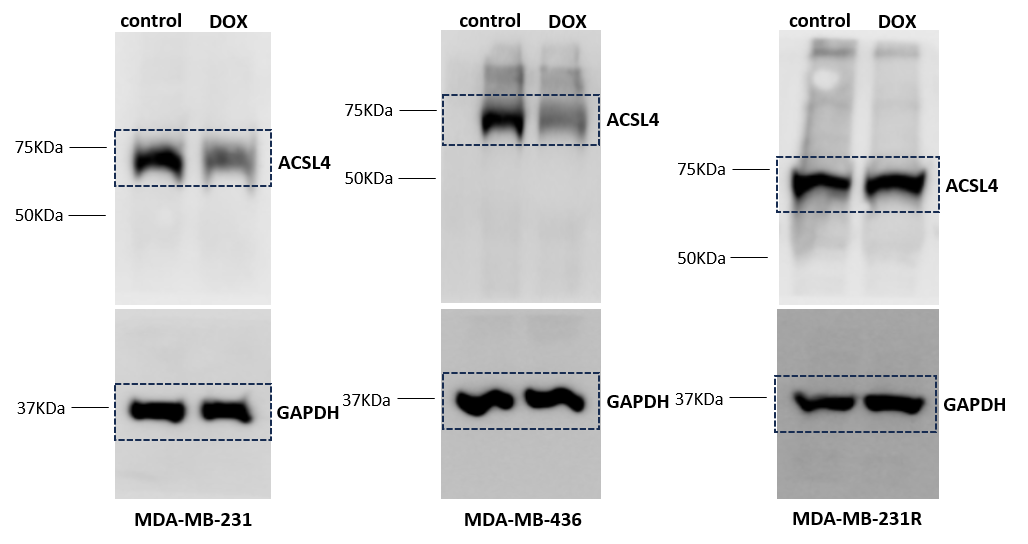


**Uncropped western blotting membranes of Figure 6B**. Abbreviations: ACSL4, acyl-CoA synthetase long-chain family member 4.


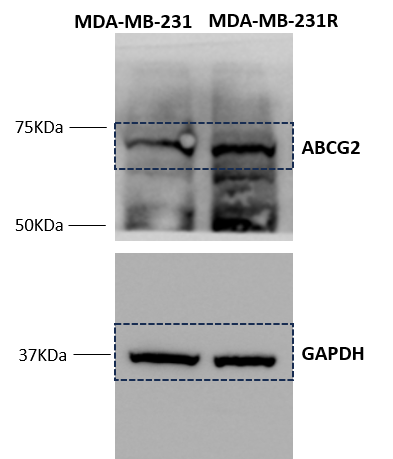


**Uncropped western blotting membranes of Figure 7B**. Abbreviations: ABCG2, ATP Binding Cassette Subfamily G Member 2.


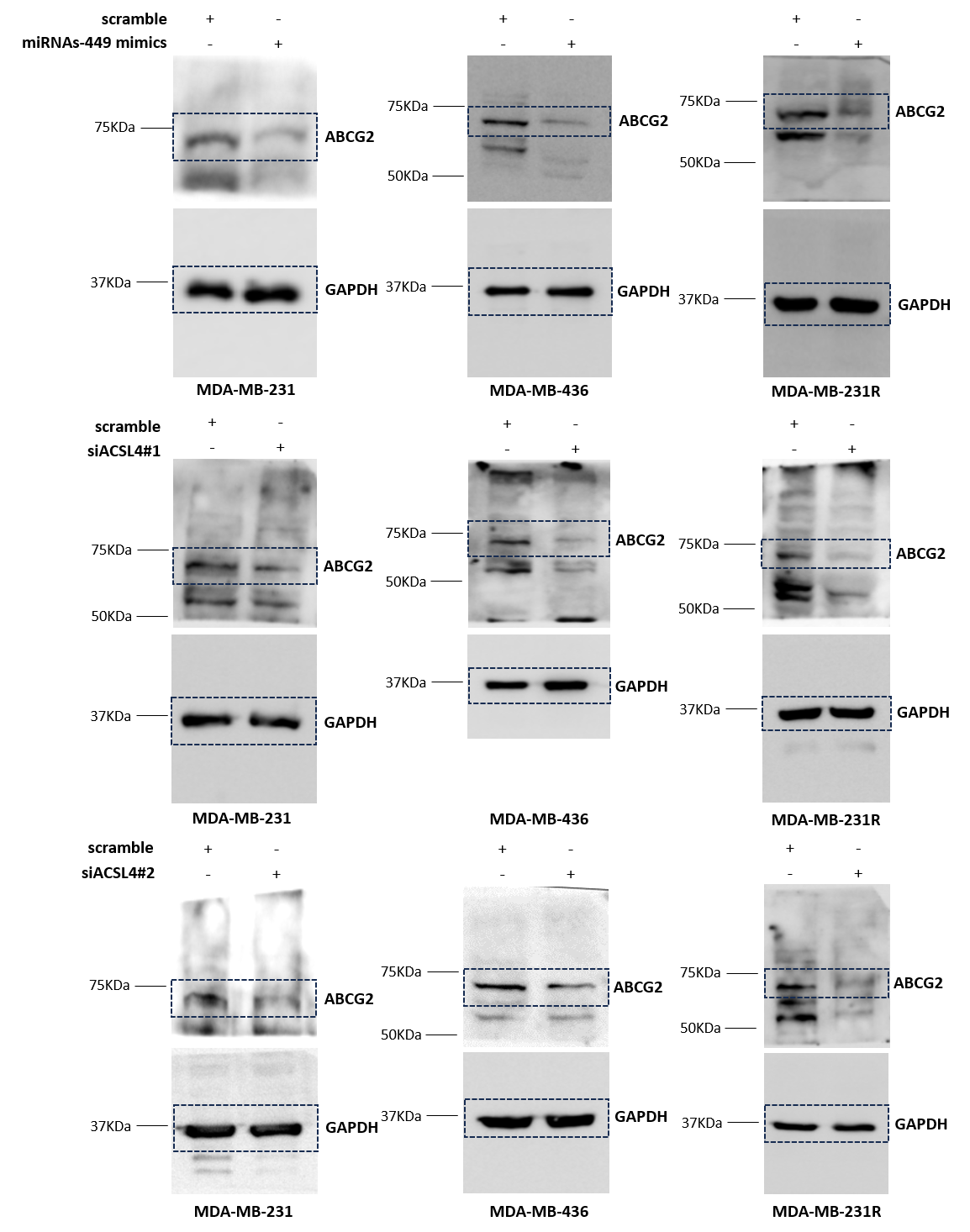


**Uncropped western blotting membranes of Figure 7C**. Abbreviations: ABCG2, ATP Binding Cassette Subfamily G Member 2.


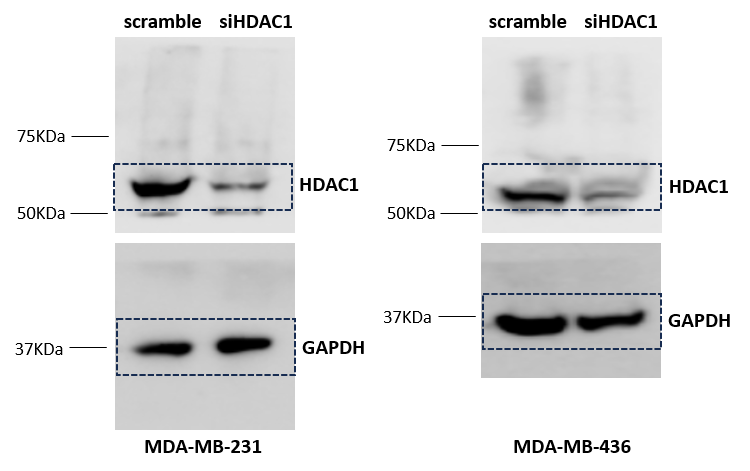


**Uncropped western blotting membranes of Figure S2B**. Abbreviations: HDAC1, histone deacetylase 1.


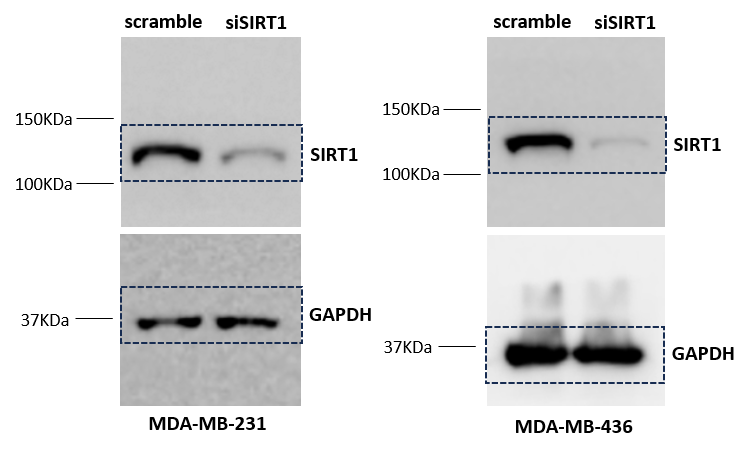


**Uncropped western blotting membranes of Figure S2D**. Abbreviations: SIRT1, sirtuin 1.


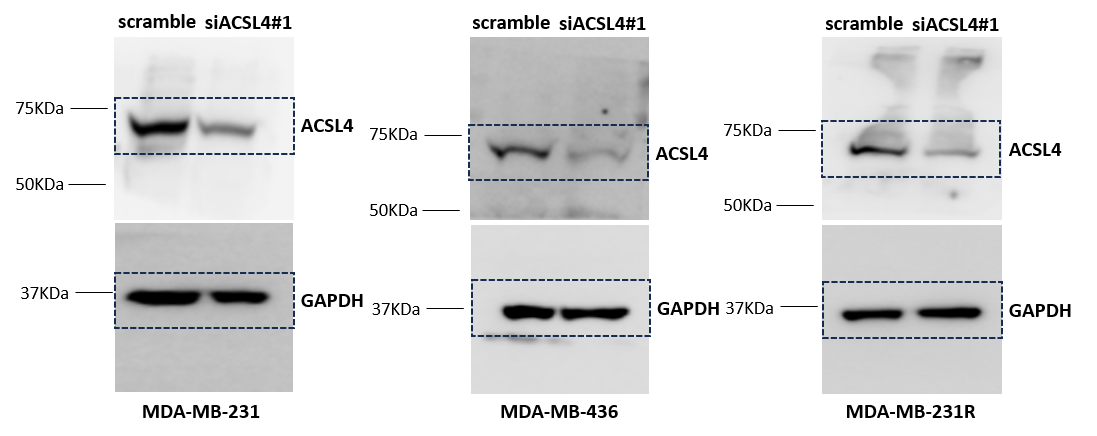


**Uncropped western blotting membranes of Figure S2K**. Abbreviations: ACSL4, acyl-CoA synthetase long-chain family member 4.


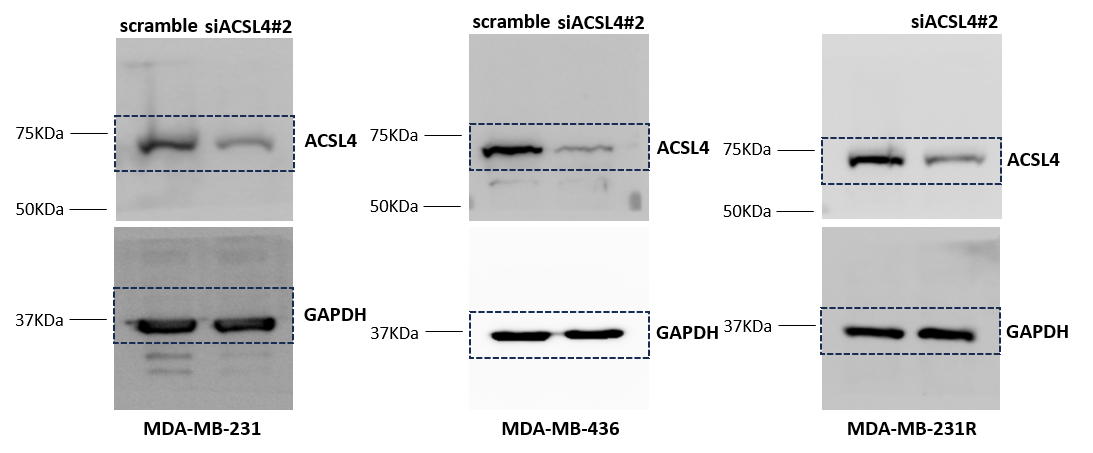


**Uncropped western blotting membranes of Figure S2L**. Abbreviations: ACSL4, acyl-CoA synthetase long-chain family member 4.
